# PIM3-mediated phosphorylation stabilizes myeloid leukemia factor 2 to promote metastasis in osteosarcoma

**DOI:** 10.1172/JCI191040

**Published:** 2025-10-15

**Authors:** Cuiling Zeng, Xin Wang, Jinkun Zhong, Yu Zhang, Ju Deng, Wenqiang Liu, Weixuan Chen, Xinhao Yu, Dian Lin, Ruhua Zhang, Shang Wang, Jianpei Lao, Qi Zhao, Li Zhong, Tiebang Kang, Dan Liao

**Affiliations:** 1 Sun Yat-sen University Cancer Center, State Key Laboratory of Oncology in South China, Guangdong Provincial Clinical Research Center for Cancer, Collaborative Innovation Center for Cancer Medicine, Guangzhou, China.; 2 Molecular Cancer Research Center, School of Medicine, Shenzhen Campus of Sun Yat-Sen University, Sun Yat-Sen University, Shenzhen, China.; 3 Shenzhen Institute of Synthetic Biology, Shenzhen Institutes of Advanced Technology, Chinese Academy of Sciences, Shenzhen, China.; 4 Center of Digestive Diseases, Scientific Research Center, The Seventh Affiliated Hospital, Sun Yat-sen University, Shenzhen, China.

**Keywords:** Cell biology, Oncology, Cancer, Protein kinases, Ubiquitin-proteosome system

## Abstract

Osteosarcoma is the most common primary malignant bone cancer, characterized by a high incidence of lung metastasis and a lack of therapeutic targets. Here, by combining an in vivo CRISPR activation screen with the interactome of STUB1, a tumor suppressor in osteosarcoma, we identified that myeloid leukemia factor 2 (MLF2) promotes osteosarcoma metastasis. Mechanistically, MLF2 disrupted the interaction between BiP and IRE1α, thereby activating the IRE1α/XBP1-S-MMP9 axis. The E3 ligase STUB1 ubiquitinated MLF2 at Lys119 and targeted it for proteasomal degradation, whereas PIM3-mediated phosphorylation of MLF2 at Ser65 enhanced its stabilizing interaction with USP21. Our findings demonstrate that the PIM3/MLF2 axis is a critical regulator of osteosarcoma lung metastasis. We propose PIM3 as a potential therapeutic target for patients with osteosarcoma lung metastasis.

## Graphical abstract

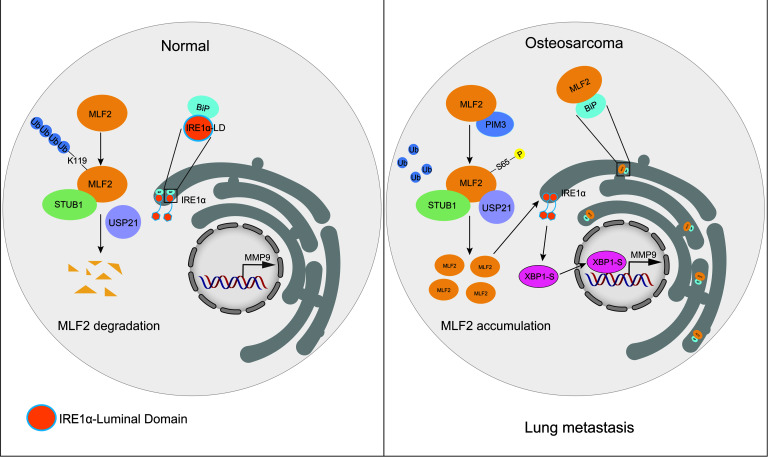

## Introduction

Osteosarcoma is among the most common primary malignant bone tumors with a high peak incidence in adolescents (1). Although the survival rate is as high as 70% after local surgery combined with neoadjuvant multidrug chemotherapy, the 5-year overall survival rate of patients with metastatic cancer is only 20%. Currently, there are no effective therapeutic targets for osteosarcoma (2, 3). A better understanding of the molecular mechanisms of osteosarcoma metastasis may thus provide therapeutic targets for patients.

The ER is a highly dynamic organelle responsible for the synthesis, folding, and quality control of secreted and transmembrane proteins. Multiple cell-intrinsic and -extrinsic stresses result in the accumulation of misfolded proteins, thereby causing ER stress (4). Under ER stress, the cells initiate an intracellular signaling pathway known as the ER stress response to restore ER homeostasis or to promote cell apoptosis if the damage is irreversible (5). The mammalian UPR is regulated by 3 transmembrane proteins localized on the ER membrane: IRE1α, ATF6, and PERK (5). In response to ER stress, IRE1α undergoes oligomerization and trans-autophosphorylation, causing a conformational change that activates its RNase domain to catalyze an unconventional splicing by removal of a 26-nucleotide intron from the XBP1 mRNA (6, 7). This splicing event leads to the translation of a functionally active transcription factor, termed XBP1-S, that promotes cell adaptation by resolving ER stress (6). Although extreme ER stress leads to a terminal UPR that induces cell death, chronic ER stress response promotes cancer cell proliferation and metastasis through the activation of angiogenesis, epithelial-mesenchymal transition, extracellular matrix remodeling, and immune evasion (5, 8).

CRISPR/Cas9 library screening has been widely used to identify functional genes related to cell viability, drug resistance, and metastasis (9–11). In this study, we performed an in vivo CRISPR activation screening for lung metastasis and found that myeloid leukemia factor 2 (MLF2), a protein of the myeloid leukemia factor (MLF) family, promotes osteosarcoma metastasis via activating the IRE1α/XBP1-S-MMP9 axis. Moreover, PIM3-mediated phosphorylation of MLF2 at Ser65 abolishes its proteasomal degradation by the E3 ligase STUB1.

## Results

### In vivo, genome-wide CRISPR activation screening identifies MLF2 as promoting lung metastasis of osteosarcoma.

**Figure 1:**
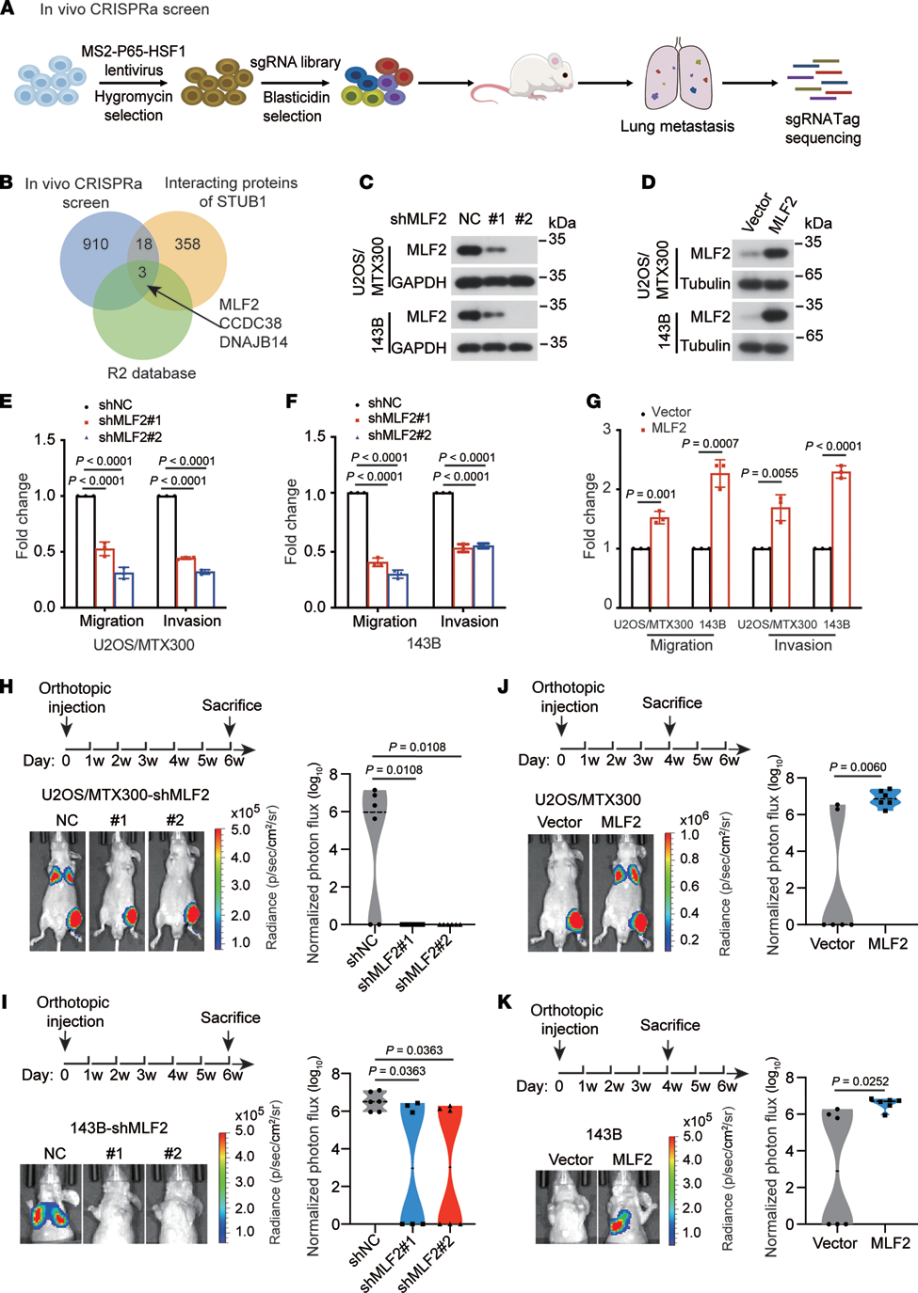
In vivo genome-wide CRISPR activation screen identifies genes promoting lung metastasis of osteosarcoma. (**A**) Diagram of in vivo CRISPR activation screening in U2OS/MTX300 cells. (**B**) Venn diagram indicates the overlaps among in vivo CRISPR activation screening, STUB1 interactome, and R2 database analysis. (**C** and **D**) Western blot analysis of MLF2 protein levels in U2OS/MTX300 and 143B cells stably expressing MLF2-targeted shRNAs or overexpression of MLF2. Data are representative of 3 independent experiments. (**E**–**G**) Quantification analyses of migration and invasion assays using the indicated U2OS/MTX300 or 143B cells stably expressing MLF2-targeted shRNAs or overexpression of MLF2. The data are presented as mean ± SD. *n* = 3 biologically independent experiments. *P* values were calculated using 1-way ANOVA with Dunnett’s test (**E** and **F**) and 2-tailed Student’s *t* test (**G**). (**H**–**K**) The procedure for the in vivo orthotopic model of osteosarcoma metastasis. Representative bioluminescence images of mice orthotopically transplanted with the indicated luciferase-transduced U2OS/MTX300 or 143B cells stably expressing MLF2-targeted shRNAs or overexpression of MLF2 (left) and quantification analyses of the metastasis of cancer cells in the lung based on the left (right). *n* = 6 mice per group. Data are presented as mean ± SD. *P* values were calculated using 2-tailed Student’s *t* test (**J** and **K**) and 2-tailed Student’s *t* test followed by Benjamini-Hochberg correction (**H** and **I**). NC, negative control; p, photon; sh, short hairpin; sr, steradian.

To explore the key tumor-promoting genes in the progression of lung metastasis in osteosarcoma, we performed an in vivo, whole-genome-wide CRISPR activation screen using the synergistic activation mediator system, which contains 70,290 sgRNAs targeting all 23,430 human genes (10). Using an orthotopic osteosarcoma lung metastasis model in vivo, U2OS/MTX300 osteosarcoma cells were infected with the lentivirus carrying dCas9 and a library of sgRNAs (Figure 1A). Two months later, the mice were sacrificed and their lung tissues were sequenced to identify the enriched sgRNAs. A small proportion of genes were significantly enriched (Supplemental Table 1; supplemental material available online with this article; https://doi.org/10.1172/JCI191040DS1). To narrow down these genes potentially promoting metastasis, we combined these data with the STUB1 IP–mass spectrometry (MS) interactome data containing 358 proteins (Supplemental Table 2), because we had recently reported that STUB1, an E3 ligase, acts as a tumor suppressor in osteosarcoma (12, 13). There were 21 genes that overlapped in these 2 data sets (Figure 1B). Then, we individually checked the clinical relevance of these genes through databases via the R2: Genomics Analysis and Visualization Platform. Three candidates—MLF2, CCDC38, and DNAJB14—were most strongly associated with poor overall survival or metastasis-free survival (Supplemental Figure 1, A–F). Knockdown of MLF2 or DNAJB14 decreased the migration and invasion of U2OS/MTX300 cells (Supplemental Figure 1, G and H). Finally, MLF2 was chosen for further investigation because it was the most significantly enriched gene in our screening.

Using U2OS/MTX300,143B, and U2OS cells, we found that knockdown of MLF2 impeded cell migration and invasion, whereas MLF2 overexpression increased these processes (Figure 1, C–G, and Supplemental Figure 2, A–D). However, depletion of MLF2 inhibited cell viability, whereas ectopic MLF2 expression had no effect (Supplemental Figure 2, E–J), indicating that MLF2 is also essential for cell viability in osteosarcoma. Consistent with these results, in an orthotopic osteosarcoma lung metastasis model with U2OS/MTX300 and 143B cells, knockdown of MLF2 reduced lung metastasis, whereas its overexpression increased lung metastasis in vivo (Figure 1, H–K, and Supplemental Figure 2, K–N). However, there was no obvious difference in primary tumor growth (Supplemental Figure 3, A–H). In addition, higher protein levels of MLF2 were detected in osteosarcoma tissues compared with adjacent normal tissues (Supplemental Figure 4, A and B). Consistently, we confirmed that mRNA levels of MLF2 were higher in tumor tissues compared with the normal tissues, through the TNMplot database (14) (Supplemental Figure 4C), and high mRNA levels of MLF2 were correlated to poor prognosis in lung cancer and gastric cancer, as analyzed by the KM Plotter database (15, 16) (Supplemental Figure 4D). Taken together, these results demonstrate that MLF2 plays a key role in osteosarcoma lung metastasis.

### MLF2 promotes osteosarcoma lung metastasis by activating the IRE1α/XBP1-S/MMP9 axis.

**Figure 2:**
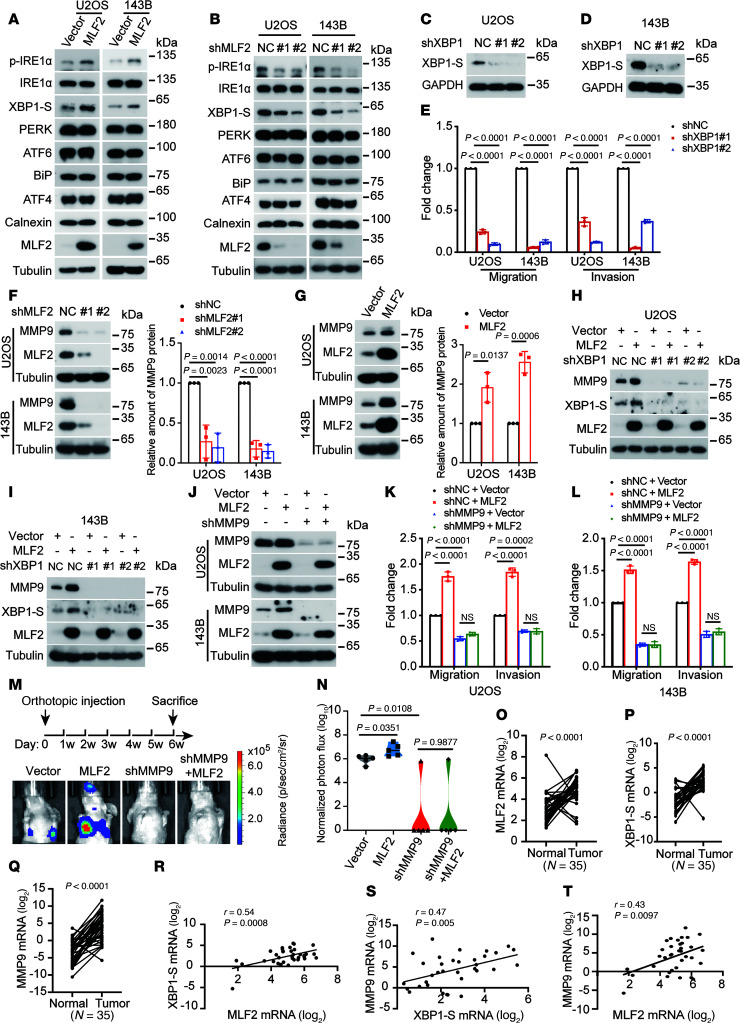
MLF2 promotes lung metastasis of osteosarcoma by activating IRE1α/XBP1-S-MMP9 axis. (**A** and **B**) The indicated proteins were analyzed by Western blotting in U2OS and 143B cells stably overexpressing MLF2 or MLF2-targeted shRNAs (**C** and **D**) Western blotting analysis of XBP1-S protein levels in U2OS and 143B cells stably expressing XBP1-targeted shRNAs. (**E**) Quantification analyses of migration and invasion assays using the indicated stable cells. (**F** and **G**) Western blotting analysis and quantification of MLF2 and MMP9 protein levels in U2OS and 143B cells stably expressing MLF2-targeted shRNAs or overexpression of MLF2. (**H** and **I**) Western blotting analysis of the indicated protein levels in indicated XBP1 knockdown cells with or without MLF2 overexpression. (**J**) Western blotting analysis of MLF2 and MPP9 protein levels in indicated MPP9 knockdown cells with or without MLF2 overexpression. (**K** and **L**) Quantification analyses of migration and invasion assays using the indicated stable cells in **J**. (**M**) The procedure for the in vivo orthotopic model of osteosarcoma metastasis and representative bioluminescence images of mice orthotopically transplanted with the indicated luciferase-transduced 143B cells. (**N**) Quantification analyses of lung metastasis based on **M**. *n* = 5 mice per group. Data are reported as mean ± SD. *P* values were calculated using 2-tailed Student’s *t* test followed by Benjamini-Hochberg correction. (**O**–**Q**) The mRNA levels of MLF2, XBP1-S, and MMP9 were analyzed by real-time PCR in human osteosarcoma tissues and adjacent normal tissues. (**R**–**T**) The correlation scatterplots are shown based on the mRNA levels of MLF2, XBP1-S, and MMP9. The coefficient of correlation and *P* value were calculated using nonparametric Spearman’s test. Data in **A**–**D** and **H**–**J** are representative of 3 independent experiments. Data in **E**–**G**, **K**, and **L** are presented as mean ± SD. *n* = 3 biologically independent experiments. *P* values were calculated using 1-way ANOVA with Dunnett’s test (**E** and **F**) or Šídák’s test (**K** and **L**) and 2-tailed Student’s *t* test (**G** and **O**–**Q**). NC, negative control; p, photon; sh, short hairpin; sr, steradian.

MLF2 is a member of the MLF family that plays an oncogenic role in breast cancer, chronic myelogenous leukemia, and colorectal cancer (17–19). To uncover the mechanism by which MLF2 promotes osteosarcoma lung metastasis, RNA-Seq analysis was performed using U2OS cells overexpressing MLF2. Gene set enrichment analysis indicated that genes upregulated upon MLF2 overexpression were enriched in the UPR, also known as ER stress response (Supplemental Figure 5, A and B). Indeed, the key genes downstream of the IRE1α/XBP1-S signaling pathway that are commonly used as ER stress response markers were increased by ectopic MLF2 in osteosarcoma cells (Supplemental Figure 5C and Figure 2A), whereas MLF2 depleting reduced them in osteosarcoma cells (Figure 2B).

Furthermore, we investigated the potential role of MLF2 in the regulation of the ER stress response in osteosarcoma. Upon thapsigargin treatment, the IRE1α/XBP1-S signaling pathway was activated by MLF2 overexpression and inhibited by MLF2 depletion in both U2OS and 143B cells (Supplemental Figure 5, D–G). In contrast, neither the PERK nor the ATF6 pathway was affected (Supplemental Figure 5, D–G). These results indicate MLF2 activates the IRE1α/XBP1-S pathway in the ER stress response.

The IRE1α/ XBP1-S pathway is critical for tumorigenesis. Knockdown of XBP1-S, a downstream effector of IRE1α, reduced the mRNA levels of XBP1-S target genes that are related to metastasis, such as MMP9 (20), PLAU, and CCND1 (Supplemental Figure 6A), and impaired cell migration and invasion (Figure 2, C–E). However, only MMP9 was reduced by MLF2 depletion and increased by MLF2 overexpression in both U2OS and 143B cells (Figure 2, F and G). Moreover, the MMP9 reporter activity was increased by overexpression of XBP1-S and decreased by knockdown of XBP1-S in U2OS cells (Supplemental Figure 6, B and C), and knockdown of XBP1-S abolished the increase in MMP9 by ectopic MLF2 in U2OS and 143B cells (Figure 2, H and I).

MMP9 depletion decreased cell migration and invasion in U2OS and 143B cells, whereas its overexpression increased these properties (Supplemental Figure 6, D–G). More importantly, MMP9 depletion abolished MLF2-induced increases in migration and invasion in U2OS and 143B cells without affecting cell viability (Figure 2, J–L, and Supplemental Figure 6H). Consistent with these results, knockdown of MMP9 in the orthotopic osteosarcoma lung metastasis model in vivo reversed the enhancement of lung metastasis induced by overexpression of MLF2 in 143B cell lines (Figure 2, M and N, and Supplemental Figure 6I). Accordingly, mRNA levels of MLF2, XBP1-S, and MMP9 were higher in osteosarcoma tissues compared with adjacent normal tissues (Figure 2, O–Q), and these levels were positively correlated in osteosarcoma tissues (Figure 2, R–T). Collectively, these results indicate MLF2 promotes osteosarcoma lung metastasis by activating the IRE1α/XBP1-S/MMP9 axis.

### MLF2 activates IRE1α/XBP1-S signaling by interfering with the interaction of BiP and IRE1α.

**Figure 3:**
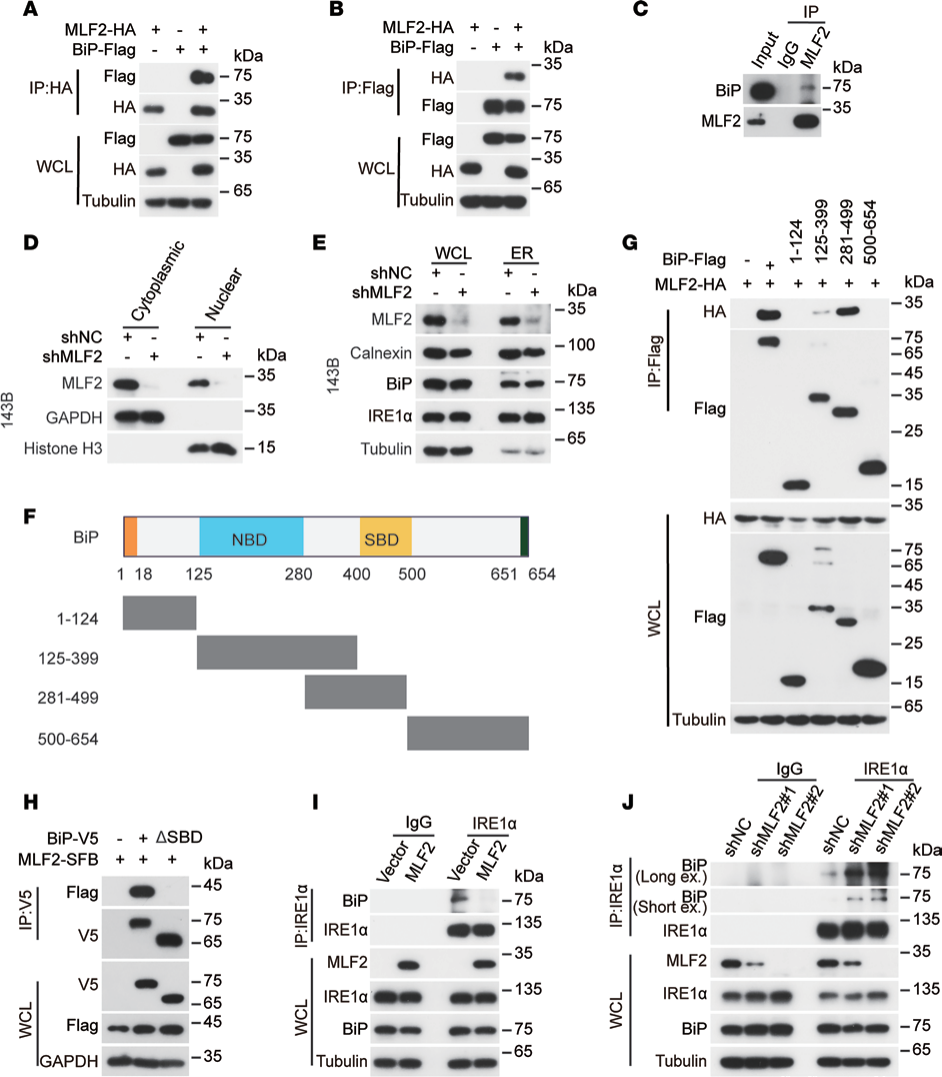
MLF2 activates IRE1α/XBP1-S signaling via interfering in the binding between BiP and IRE1α. (**A** and **B**) HEK293T cells were cotransfected with MLF2-HA and BiP-Flag for 48 hours and then subjected to IP using anti-FLAG antibody (**A**) or anti-HA antibody (**B**) followed by Western blotting analysis. (**C**) The endogenous interaction between MLF2 and BiP in 143B cells was measured by co-IP with anti-MLF2 antibody, and isotype-matched IgG was used as a control. (**D**) The nuclear and cytoplasmic locations of MLF2 in 143B cells were analyzed by the NE-PER Nuclear and Cytoplasmic Extraction Kit. (**E**) The ER location of MLF2 in 143B cells was analyzed with the ER enrichment kit. (**F**) Schematic illustration of BiP structure. (**G** and **H**) The domain structure of BiP, which interacts with MLF2, was measured by co-IP. HEK293T cells were cotransfected with the indicated plasmids for 48 hours and then subjected to IP using anti-FLAG antibody (**G**) or anti-V5 antibody (**H**), followed by Western blotting analysis. (**I** and **J**) Lysates from U2OS cells stably overexpression of MLF2 (**I**) and 143B cells stably expressing MLF2-targeted shRNAs (**J**) were subjected to IP using anti-IRE1α antibody or anti-IgG antibody followed by Western blotting, as indicated. Data in **A**–**E** and **G**–**J** are representative of 3 independent experiments. NC, negative control; sh, short hairpin; NBD, nucleotide-binding domain; SBD, substrate-binding domain; WCL, whole cell lysate.

We next investigated how MLF2 activates the ER stress response. Tandem affinity purification (TAP) MS (TAP-MS) was performed to identify the MLF2 interaction partners (Supplemental Table 3) involved in the ER stress response. Notably, binding immunoglobulin protein (BiP) (also known as GRP78), an ER chaperone crucial for ER stress response (21, 22), was listed as an MLF2 binding protein. MLF2 did have a strong colocalization with BiP (Supplemental Figure 7A): the interaction between MLF2 and BiP was detected at endogenous and exogenous levels (Figure 3, A–C). To determine the subcellular localization of MLF2, we isolated cytoplasmic and nuclear fractions, and found MLF2 present in both compartments (Figure 3D and Supplemental Figure 7B).

Given that BiP is mainly located in the ER lumen, due to the presence of an ER signaling peptide (23), and MLF2 strongly colocalizes with the ER marker calnexin (Supplemental Figure 7C), we speculated that MLF2 might translocate to the ER lumen to activate IRE1α/XBP1-S signaling. To verify this hypothesis, we isolated, using the ER Enrichment Kit, the ER fraction from cells stably depleted of MLF2 and found that MLF2, Bip, IRE1α, and calnexin were enriched in the ER fraction (Figure 3E and Supplemental Figure 7D). BiP is released from the ER stress sensor IRE1α upon cellular stress to activate the ER stress IRE1α/XBP1-S signaling (24). We found that the substrate-binding domain (SBD), but not the nucleotide-binding domain, of BiP was required for its interaction with MLF2 (Figure 3, F–H). Moreover, the interaction of IRE1α with BiP at the endogenous level was blocked by ectopic MLF2 and augmented by depleting MLF2 (Figure 3, I and J). These results suggest MLF2 may act as an unfolded protein to activate IRE1α/XBP1-S signaling by interfering with the interaction of BiP with IRE1α.

### STUB1 is an E3 ligase responsible for ubiquitination and degradation of MLF2.

**Figure 4:**
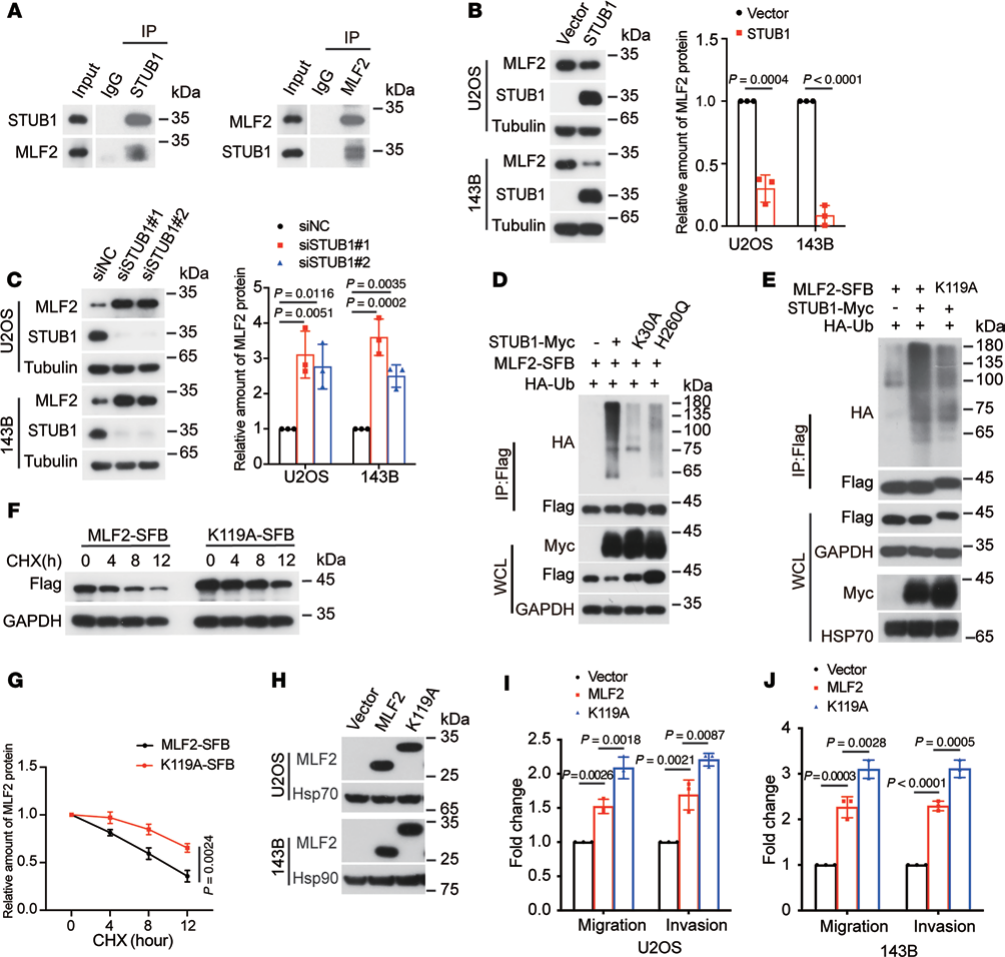
STUB1 is the E3 ligase responsible for the ubiquitination and degradation of MLF2. (**A**) The endogenous interaction between MLF2 and STUB1 in U2OS cells was measured by co-IP with anti-MLF2 antibody and anti-STUB1 antibody; isotype-matched IgG was used as a control. (**B** and **C**) The expression levels of STUB1 and MLF2 were analyzed by Western blotting in U2OS and 143B cells transfected with STUB1 or STUB1-targeted siRNAs. Quantification of MLF2 protein levels was based on the Western blotting results. (**D** and **E**) HEK293T cells were cotransfected with the indicated plasmids for 48 hours and then subjected to IP using anti-FLAG antibody followed by Western blotting. (**F** and **G**) HEK293T cells transfected with MLF2-SFB or K119A-SFB for 36 hours were incubated with 40 μg/mL cycloheximide (CHX) for the indicated periods and then analyzed by Western blotting (**F**). Quantitation of MLF2 protein levels was based on the Western blotting results (**G**). (**H**) The expression levels of MLF2 were analyzed by Western blotting in U2OS and 143B cells stably overexpression of MLF2 or MLF2 K119A mutant. (**I** and **J**) Quantification analyses of migration and invasion assays using the indicated U2OS and 143B stable cells in **H**. Data in **A**, **D**–**F**, and **H** are representative of 3 independent experiments. Data in **B**, **C**, **G**, **I**, and **J** are presented as mean ± SD. *n* = 3 biologically independent experiments. *P* values were calculated using 2-tailed Student’s *t* test (**B** and **G**) and 1-way ANOVA with Dunnett’s test (**C**) or Tukey’s test (**I** and **J**). NC, negative control; Ub, ubiquitin.

Because MLF2 may act as an unfolded protein to activate the ER stress response, as just described, it is predicated to be an unstable protein. This proved to be the case. Proteasome inhibitor MG132 markedly increased endogenous MLF2 protein levels (Supplemental Figure 8A). Therefore, the E3 ligase STUB1, identified as an interacting protein of MLF2 by TAP-MS, caught our attention. STUB1 has recently been reported by our group to be a tumor suppressor in osteosarcoma (12). The interaction of MLF2 with STUB1 was detected by co-IP at exogenous and endogenous levels (Supplemental Figure 8, B and C, and Figure 4A). The tetratricopeptide repeat domain of STUB1 was crucial for the interaction with MLF2 (Supplemental Figure 8, D–F), and the K30A mutant within the tetratricopeptide repeat domain of STUB1, but not the H260Q mutant that lacked E3 ligase activity, completely abolished binding to MLF2 (Supplemental Figure 8G). STUB1 overexpression decreased MLF2 protein levels, whereas STUB1 knockdown increased MLF2 protein levels in U2OS and 143B cells (Figure 4, B and C), and WT STUB1, but neither its K30A mutant nor H260Q mutant, was able to polyubiquitinate MLF2 (Figure 4D).

The ubiquitination usually occurs on lysine residues (25, 26), and there is only 1 lysine residue (K119) within MLF2. STUB1 could increase the polyubiquitination of the WT MLF2 but not the K119A mutant (Figure 4E). Consistently, the K119A mutant of MLF2 exhibited a longer half-life than WT MLF2 (Figure 4, F and G). Additionally, stable expression of the K119A mutant of MLF2 augmented migration and invasion in both U2OS and 143B cells compared with WT MLF2 overexpression (Figure 4, H–J). Collectively, these results indicate STUB1 polyubiquitinates MLF2 at K119.

### PIM3 stabilizes MLF2 by phosphorylating MLF2 at ser65.

**Figure 5:**
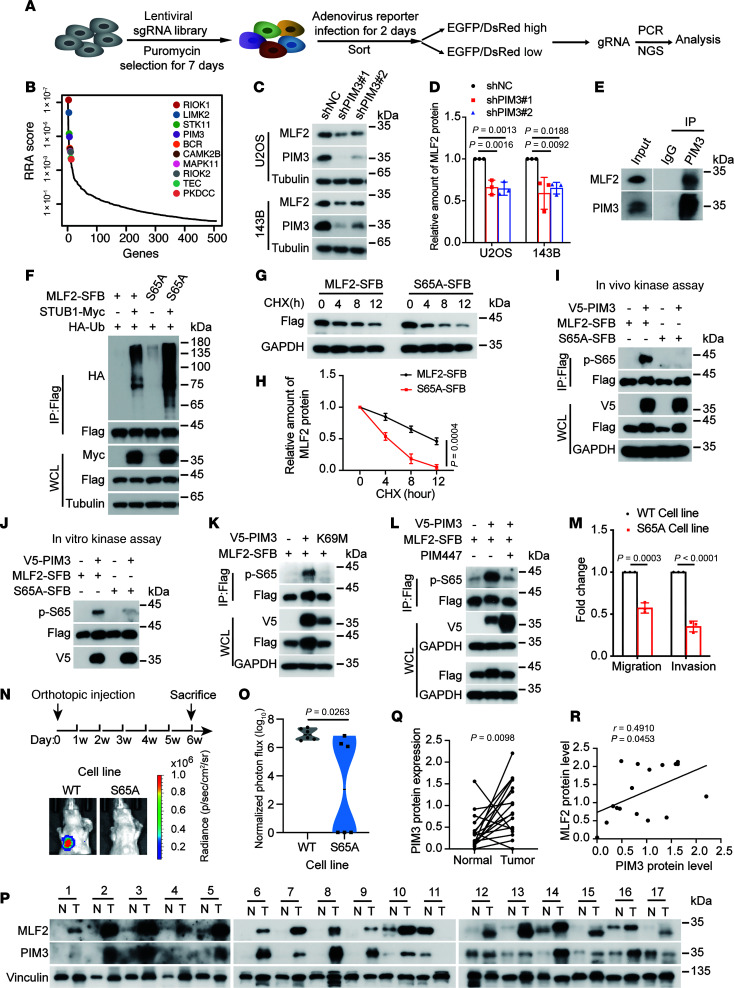
PIM3 stabilizes MLF2 by phosphorylating MLF2 at S65. (**A**) Schematic of MLF2 ProSRSA. (**B**) Top 10 candidate genes of MAGeCK analysis. (**C** and **D**) Western blotting analysis and quantification of MLF2 and PIM3 protein levels in U2OS and 143B stable cells. (**E**) 143B cells were subjected to co-IP using anti-PIM3 or anti-IgG to detect endogenous MLF2. (**F**, **I**, and **K**) HEK293T cells were cotransfected with the indicated plasmids for 48 hours and then subjected to IP assay. (**G** and **H**) HEK293T cells transfected with MLF2-SFB or S65A-SFB for 36 hours were incubated with 40 μg/mL cycloheximide (CHX) for the indicated periods and then subjected to Western blotting. Quantification of MLF2 protein levels was based on the Western blotting results. (**J**) Purified MLF2-SFB WT or S65A mutant protein were incubated with or without purified V5-PIM3 protein in vitro as described in the methods and then analyzed by Western blotting. (**L**) HEK293T cells cotransfected with MLF2-SFB and V5-PIM3 for 24 hours were incubated with PIM447 (10 μM) for another 24 hours and then subjected to IP assay. (**M**) Quantification analyses of migration and invasion assays using MLF2 WT or S65A knock-in 143B cells. (**N**) The procedure for in vivo orthotopic model of osteosarcoma metastasis and representative bioluminescence images of mice. (**O**) Quantification analyses of lung metastasis (*n* = 6). (**P** and **Q**) Western blotting analysis and quantification of MLF2 and PIM3 protein levels in human osteosarcoma tissues. N, normal; T, tumor. (**R**) The correlation between MLF2 and PIM3 protein levels is shown. The coefficient of correlation and *P* value were calculated using nonparametric Spearman’s test. Data in **C**, **E**–**G**, and **I**–**L** are representative of 3 independent experiments. Data in **D**, **H**, and **M** are presented as mean ± SD; *n* = 3 biologically independent experiments. *P* values were calculated using 1-way ANOVA with Dunnett’s test (**D**) and 2-tailed Student’s *t* test (**H**, **M**, **O**, and **Q**). NC, negative control; sh, short hairpin; sr, steradian; RRA, robust rank aggregation.

Protein degradation and stability are usually regulated by phosphorylation (27, 28). To explore the kinases that may regulate MLF2 protein stability, we performed a protein stability regulators screening assay (ProSRSA) with the CRISPR-Cas9 kinase library using U2OS cells expressing DsRed-IRES-EGFP-MLF2 reporter (29) (Figure 5A). Among the top 10 candidates identified via Model-Based Analysis of Genome-wide CRISPR/Cas9 Knockout (MAGeCK) analysis, PIM3, MAPK11, and RIOK2 were confirmed to be the potential kinases for the stabilization of MLF2 protein, because cotransfecting each of them with MLF2 elevated the MLF2 protein level, with PIM3 showing the most marked effect (Figure 5B and Supplemental Figure 9, A and B).

That MLF2 interacted most strongly with PIM3 (Supplemental Figure 9A) prompted us to focus on PIM3 for further investigation. Knockdown of PIM3 decreased MLF2 protein levels, as well as cell migration and invasion, in U2OS and 143B cells, whereas overexpression of PIM3 increased these variables (Figure 5, C and D, and Supplemental Figure 9, C–F). In addition, mRNA levels of PIM3 were higher in osteosarcoma tissues compared with corresponding normal tissues (Supplemental Figure 9G), and high PIM3 mRNA levels trended toward lower overall survival, although this association did not reach statistical significance (Supplemental Figure 9H). These results indicate PIM3 may stabilize MLF2, allowing it to play an oncogenic role in osteosarcoma.

In addition, the interaction of MLF2 with PIM3 was validated at the ectopic and endogenous levels (Supplemental Figure 10, A and B, and Figure 5E), and PIM3 was able to phosphorylate serine and threonine of MLF2 at exogenous levels (Supplemental Figure 10C). To identify the phosphorylation site(s) of MLF2 by PIM3, all serine and threonine residues of MLF2 were individually mutated to alanine. Among these mutants, only S65A and T101A mutants of MLF2 were resistant to PIM3 (Supplemental Figure 10, D and E). However, ubiquitination of the S65A mutant, but not the T101A mutant, was enhanced compared with the WT MLF2 (Figure 5F and Supplemental Figure 10F), indicating that PIM3-mediated phosphorylation at Ser65 suppresses MLF2 polyubiquitination. Moreover, the mutation of S65A shortened the half-life of MLF2 compared with the WT (Figure 5, G and H). Using an antibody that specifically recognized the Ser65 phosphorylation of MLF2, the phosphorylation of MLF2, but not its S65A mutant, was increased by PIM3 in cells and in an in vitro kinase assay (Figure 5, I and J). Furthermore, the kinase-dead mutant (K69M) of PIM3 (30, 31) and PIM inhibitor PIM447 (32, 33) reduced the phosphorylation of MLF2 at Ser65 (Figure 5, K and L).

PIM3 belongs to the highly conserved PIM family of kinases, which includes PIM1, PIM2, and PIM3 (34). Compared with PIM1 and PIM2, PIM3 had strongest binding to MLF2 and phosphorylated MLF2 Ser65 to the greatest extent (Supplemental Figure 10, G and H). Using CRISPR-Cas9–mediated homology-directed repair (35), we generated a locus-specific S65A knock-in 143B cell line (which we refer to as S65A cells) (Supplemental Figure 10I). Compared with WT cells, the half-life of MLF2 was shortened and its polyubiquitination was increased in S65A cells (Supplemental Figure 10, J–L). Moreover, although the cell viability remained unchanged in S65A cells, their migration, invasion, and lung metastasis were decreased (Figure 5, M–O, and Supplemental Figure 10, M and N). In addition, higher protein levels of PIM3 were detected in osteosarcoma tissues compared with adjacent normal tissues, and a positive correlation was observed between the protein levels of PIM3 and MLF2 in osteosarcoma tissues (Figure 5, P–R). Together, our results reveal that PIM3 stabilizes MLF2 by phosphorylating MLF2 at ser65 in osteosarcoma.

### USP21 deubiquitylates MLF2 to regulate MLF2 protein stability.

**Figure 6:**
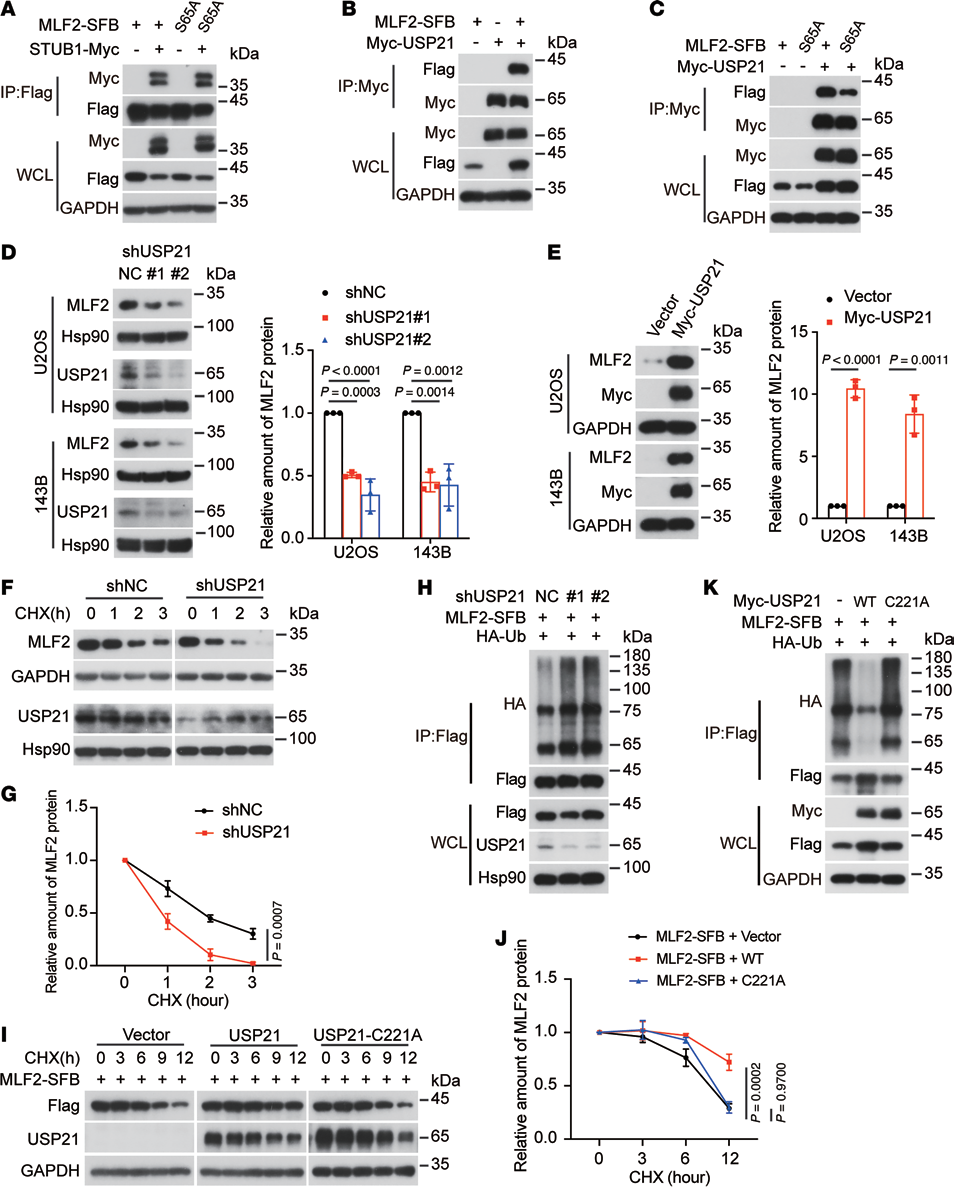
The phosphorylation of MLF2 at S65 contributed to its binding with USP21. (**A**–**C**) HEK293T cells were cotransfected with the indicated plasmids for 48 hours and then subjected to IP using anti-FLAG antibody or anti-Myc antibody, followed by Western blotting. (**D** and **E**) Western blotting analysis and quantification of MLF2 and USP21 protein levels in the indicated U2OS or 143B cells stably expressing USP21-targeted shRNAs or overexpressing USP21. (**F** and **G**) The indicated U2OS stable cells with or without USP21 knockdown were incubated with 20 μg/mL cycloheximide (CHX) for the indicated periods and then analyzed by Western blotting (**F**). Quantitation of MLF2 protein levels was based on the Western blotting results (**G**). (**H**) U2OS cells stably expressing USP21-targeted shRNAs were cotransfected with MLF2-SFB and HA-ubiquitin (HA-Ub) for 48 hours and then subjected to IP using anti-FLAG antibody followed by Western blotting. (**I** and **J**) HEK293T cells cotransfected with MLF2-SFB and vector, USP21, or mutant USP21 (C221A) for 36 hours were incubated with 40 μg/mL CHX for the indicated periods and then analyzed by Western blotting (**I**). Quantitation of MLF2 protein levels was based on the Western blotting results (**J**). (**K**) HEK293T cells were cotransfected with the indicated plasmids for 48 hours and then subjected to IP using anti-FLAG antibody followed by Western blotting. Data in **A**–**C**, **F**, **H**, **I**, and **K** are representative of 3 independent experiments. Data in **D**, **E**, **G**, and **J** are presented as mean ± SD. *n* = 3 biologically independent experiments. *P* values were calculated using 1-way ANOVA with Dunnett’s test (**D** and **J**) and 2-tailed Student’s *t* test (**E** and **G**). NC, negative control; sh, short hairpin.

Next, we investigated how the phosphorylation of MLF2 at Ser65 influences its stability. Relative to WT MLF2, the S65A mutant markedly enhanced STUB1-mediated ubiquitination and degradation, yet its interaction with STUB1 remained unchanged (Figure 6A). Ubiquitin modification is a reversible process regulated by the enzyme ubiquitin ligase that can connect ubiquitin molecules to a lysine of the substrate protein (25), whereas deubiquitylases (DUBs) can remove ubiquitin molecules from substrates (36). Thus, we sought to determine whether the S65A mutant of MLF2 would affect its binding to DUBs. However, we found that USP11, reported to be a DUB of MLF2 (37), could bind equally well to both WT MLF2 and the S65A mutant (Supplemental Figure 11A). Therefore, we performed a screening using the 22 DUBs library to identify the DUB involved in the degradation of MLF2 in osteosarcoma. Interestingly, USP21 was the most marked DUB shown to stabilize the protein levels of MLF2 (Supplemental Figure 11, B and C). The interaction of MLF2 with USP21 was confirmed at the exogenous level (Figure 6B and Supplemental Figure 11D). Importantly, interaction of USP21 with the S65A mutant was decreased compared with the WT (Figure 6C). USP21 depletion reduced MLF2 protein levels, whereas USP21 overexpression increased MLF2 protein levels in U2OS and 143B cells (Figure 6, D and E). Indeed, knockdown of USP21 shortened the half-life and increased the ubiquitination of MLF2 (Figure 6, F–H). Conversely, the WT USP21, but not its C221A inactive mutant, prolonged the half-life and reduced the ubiquitination of MLF2 (Figure 6, I–K). Taken together, these results suggest phosphorylation of MLF2 at Ser65 by PIM3 augments its interaction with USP21, resulting in the stabilization of MLF2.

## Discussion

**Figure 7:**
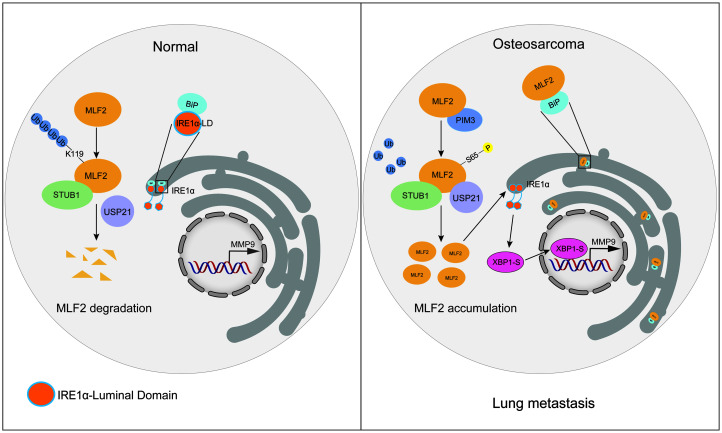
A proposed model for both function and regulation of MLF2 in osteosarcoma. In the normal condition, MLF2 can be polyubiquitinated at lysine119 by STUB1, leading to its proteasome degradation. In osteosarcoma, this process is abolished by PIM3 via phosphorylation of MLF2 at Ser65, which enhances its binding with USP21. MLF2 stabilization activates IRE1α/XBP1-S-MMP9 axis via interfering the interaction between IRE1α and BiP, thereby promoting osteosarcoma lung metastasis.

In this study, we found that under normal conditions, MLF2 can be polyubiquitinated at lysine119 by STUB1, leading to its proteasome degradation (Figure 7). PIM3 is overexpressed in osteosarcoma, and it can phosphorylate MLF2 at Ser65 to enhance its interaction with USP21. Consequently, the MLF2 protein is stabilized and is able to promote osteosarcoma lung metastasis via activating the ER stress response.

MLF2 shares about 40% identity with its homolog MLF1 (38, 39). Compared with MLF1, the localization and function of MLF2 are poorly documented. Recently, it has been reported that MLF2 is located in the nucleus, where it interacts with and negatively regulates p53 to promote colorectal carcinogenesis (19). Here, we found that MLF2 is primarily located in the cytoplasm and can translocate to the ER to promote osteosarcoma lung metastasis via interacting with BiP, although MLF2 was distributed in both the nucleus and cytoplasm. These results indicate that, similar to MLF1, MLF2 may be a nucleocytoplasmic shuttling protein, and it may have different functions depending on its cellular compartment. How its cellular location is regulated remains an open question for future investigation.

The ER stress response plays a role in metastasis by regulating the adaptation of tumor cells to their microenvironment (4, 5). For example, key molecules related to cancer metastasis, such as collagens, MMPs, and integrins, are regulated by the UPR (40). Here, we report that MLF2, as an unstable protein, can activate the IRE1α/XBP1-S/MMP9 axis and thereby promote lung metastasis of osteosarcoma. Accordingly, we found the level of XBP1-S was higher in osteosarcoma tissues than in normal tissues, and that XBP1-S promotes cell migration and invasion in osteosarcoma. These results are consistent with reports in the literature: XBP1 has been reported to be overexpressed in osteosarcoma tissues and involved in tumor progression (41), and XBP1-S has also been shown to be overexpressed and positively correlated with poor prognosis in breast cancer (42), myeloma (43), glioblastoma (44), and pulmonary adenocarcinoma (44).

Upon ER stress, the accumulation of unfolded proteins in the ER causes the dissociation of BiP from ER stress sensors, resulting in activation of the ER stress response (4, 5). There are 2 main models used to describe the ER stress-sensing mechanism by IRE1α and PERK: a direct recognition model in which IRE1α is activated by binding of unfolded proteins to the luminal domains of IRE1α (45), and an indirect model in which the ER stress-sensing process is directly coupled to the folding machinery. In the indirect model, BiP is responsible for sensing unfolded proteins, and the release of BiP from IRE1α is the key process involved in the activation of IRE1α (21, 22, 24, 46). Here, we report that MLF2 can interact with BiP to release BiP from IRE1α, suggesting that MLF2 may function as an unfolded protein that activates IRE1α.

The E3 ligase CRL4^DCAF8^ can promote MLF2 ubiquitination and degradation (37); however, CRL4^DCAF8^ was not identified among the proteins interacting with MLF2 by TAP-MS. Instead, we found that STUB1 was responsible for the ubiquitination and degradation of MLF2, reinforcing the suggestion that STUB1 may act as a tumor suppressor in osteosarcoma. This is supported by our recent reports showing that STUB1 functions by degrading several substrates, including Rab22a-Neof1 fusion protein (12), CBX4 (13), and IRS4 (47).

We also showed that PIM3, which belongs to the PIM kinase family that includes PIM1, PIM2, and PIM3, is upregulated and promotes cell migration and invasion in osteosarcoma. This finding is consistent with reports in the literature showing that PIM3 is upregulated and acts as an oncogene in several cancers (48–51), and that PIM kinases are frequently expressed in osteosarcoma. More importantly, we identified MLF2 as a new substrate for PIM3 in osteosarcoma. Phosphorylation of MLF2 at Ser65 by PIM3 enhances its binding to USP21, thereby stabilizing the MLF2 protein. On the basis of these findings, we propose the PIM3-MLF2 axis as a potential therapeutic target for patients with osteosarcoma lung metastasis.

## Methods

### Sex as a biological variable.

In this study, sex was not considered as a biological variable. All mice used in this study were male.

### Cell culture.

The HEK293T, U2OS, and 143B cells were obtained from the American Type Culture Collection (ATCC) and cultured according to the instructions from the ATCC. The U2OS/MTX300 cell line was an methotrexate-resistant (MTX-resistant) variant (MTX: 300 ng/mL) derived from the U2OS cell line and was cultured as previously described (52, 53). The 143B-Luc and U2OS/MTX300-Luc cells stably expressing luciferase were cultured as previously described (53). All cell lines were cultured in DMEM supplemented with 10% FBS at 37°C and 5% CO_2_. All cell lines used in this study were authenticated using short-tandem-repeat profiling less than 6 months before the project was initiated, and cells were not cultured for longer than 1 month.

### Plasmids.

The cDNAs of MLF2, MMP9, BiP, and PIM3 were amplified by PCR and cloned into a PSIN vector. The Myc-tagged STUB1 and USP21, HA-tagged MLF2, Flag-tagged BiP, and V5-tagged PIM3 were cloned into the pCDNA3.1 vector. The promoter regions of MMP9 were cloned into the pGL3-basic vector. The shRNAs targeting MLF2, XBP1, MMP9, and PIM3 were cloned into the PLKO.1-puro vector. The sequences used for the indicated shRNAs are provided in Supplemental Table 6.

### In vivo CRISPR activation screen.

A total of 1.2 × 10^8^ U2OS/MTX300 cells stably expressing luciferase and MS2-P65-HSF1 (Addgene, 89308) were transduced with the human CRISPR/Cas9 SAMv2 pooled library (Addgene, 1000000078) at an MOI of 0.3, as previously described (10). The library representation was greater than 500X and was selected with blasticidin at 12.5 μg/mL for 7 days. Immediately after selection, 3.6 × 10^7^ of these cells were transplanted orthotopically into the bones of 18 BALB/c nude mice, with 2 × 10^6^ cells each, as previously described (54, 55). At the same time, DNA from 3.6 × 10^7^ cells (500 cells/sgRNA) was isolated as an input baseline distribution of sgRNAs. The mice were monitored for lung metastasis using the IVIS Lumina System (PerkinElmer). After 2 months, lung metastasis was observed in 9 of 18 mice. All mice were sacrificed and the whole lungs from mice with metastasis were harvested. The lungs were divided into 25 μg chunks and homogenized using a tissue homogenizer, and genomic DNA was isolated using a DNA Extraction Kit (Qiagen) according to the manufacturer’s protocol. The sgRNAs were amplified using NEBNext High Fidelity 2X Master Mix (New England Biolabs); the primer sequences are detailed in Supplemental Table 6. The resulting PCR products from all reactions were pooled and purified using a PCR Purification Kit (Tiangen), in preparation for sequencing on the Illumina NovaSeq 6000 platform.

### Screen analysis.

The screen analysis was conducted, with slight modifications, based on an article by Ebright et al. (56). sgRNA represented with fewer than 50 sequencing reads in lung tissues were excluded for analysis. sgRNA read counts for each sample were normalized to the total counts for that sample. sgRNA distribution for each mouse was compared with the input distribution of sgRNAs, resulting in a fold change value for each sgRNA for each mouse. Fold change was averaged across all mice to yield an average fold change for each sgRNA; the most enriched sgRNA for each gene was determined, and corresponding genes were rank ordered based on their average fold change. The enrichment results and row read counts for all samples are listed in Supplemental Table 1.

### CRISPR-Cas9 Screening for the MLF2 ProSRSA.

This procedure has been described (29). Briefly, 2 × 10^7^ U2OS cells were infected with the CRISPR-Cas9 kinase library at an MOI of 0.3, with a guide representation of 500 cells/guide. Forty-eight hours after transfection, the cells were selected with 0.5 μg/mL puromycin for 7 days. Then the cells were reinfected with pAd-DsRed-IRES-EGFP-MLF2 adenovirus at an MOI of 4.0 to ensure that greater than 95% of the cells were positive. Forty-eight hours later, the cells were collected and sorted into EGFP/DsRed high (5%) and EGFP/DsRed low (5%) populations using a Beckman Coulter MoFlo Cell Sorting System. Genomic DNA was isolated from EGFP/DsRed high and EGFP/DsRed low cells using a TIANamp genomic DNA kit according to the manufacturer’s protocol (Tiangen). The sgRNAs were amplified using primers detailed in Supplemental Table 6. The resulting PCR products were pooled and purified using a PCR Purification Kit (Tiangen) in preparation for Illumina NovaSeq 6000 sequencing. Raw sequence data were trimmed to 20 bp by removing a constant portion of the sgRNA sequences and then were analyzed with the MAGeCK package. The full results are reported in Supplemental Table 5.

### RNA-Seq.

U2OS cells stably expressing MLF2 and vector were collected, and total RNA was extracted using TRIzol reagent (Life Technologies, Thermo Fisher Scientific, 15596026). RNA-Seq was performed by Novogene using the Illumina NovaSeq 6000 platform. A total of 6 GB of clean data per sample were collected for RNA-Seq, and the resultant clean reads were aligned to the human genome GRCh38 (Hg38) using Spliced Transcripts Alignment to a Reference (STAR) aligner. The edgeR algorithm was applied to filter the differentially expressed genes based on the following criteria: |log2 fold change| > 1 and a *P* value <0.05. The results of differential gene expression between vector and MLF2 are reported in Supplemental Table 3.

### MS analysis.

Affinity purification was carried out for cells overexpressing STUB1-SFB and MLF2-SFB. HEK293T cells stably expressing STUB1-SFB and U2OS cells stably expressing MLF2-SFB were lysed in NETN buffer (100mM Nacl, 20 mM Tris-Cl, pH 8.0, 0.5 mM EDTA, 0.5 % NP-40). The lysates were centrifuged at 15,294*g*, and the supernatants were incubated with streptavidin-conjugated beads overnight at 4°C. The beads were washed 5 times with NETN buffer and then eluted with NETN buffer containing 2 mg/mL biotin (MilliporeSigma, Merck) for 6 hours. The elutes were incubated with S-protein beads (Novagen, Sigma-Aldrich) overnight at 4°C. The beads were washed 5 times, and the bound proteins were analyzed by SDS-PAGE. MS was performed by APTBIO (Shanghai Applied Protein Technology).

### RNAi treatment.

The siRNAs targeting specific genes were designed and synthesized by RiboBio. Transfection was performed according to the manufacturer’s instructions using Lipofectamine RNAiMAX transfection reagent (Invitrogen, Thermo Fisher Scientific) and 50 nmol/L siRNA. The target sequences of siRNAs we used are listed in the Supplemental Table 6.

### RNA extraction and qRT-PCR.

Total RNA was isolated using an RNA extraction kit (Tiangen), and cDNA was synthesized using a HiScript 1st Strand cDNA Synthesis Kit (Vazyme Biotech) according to the manufacturer’s instructions. qRT-PCR was performed using a Light Cycler 480 instrument (F. Hoffmann-La Roche) with 2X SYBR Green mix (Vazyme International). All reactions were carried out in triplicate in a 10 μL reaction volume. Standard curves were generated, and the relative amount of target-gene mRNA was normalized to that of GAPDH. The primers used for qRT-PCR are listed in the Supplemental Table 6.

### The luciferase reporter assay.

Briefly, the cells were seeded in 24-well plates and then were cotransfected with 250 ng of promoter-luciferase plasmid and 5 ng of pRL-TK (*Renilla* luciferase) control vector. After transfection for 36 hours, the luciferase activity was measured using a Dual-Luciferase Assay Kit (Promega) according to the manufacturer’s instructions. Firefly luciferase activity was normalized to *Renilla* luciferase activity for each sample. Three independent experiments were performed, and the calculated means and SDs are presented.

### Immunofluorescence.

U2OS cells were washed 3 times with PBS and fixed in 4% paraformaldehyde for 15 minutes, followed by permeabilization with 0.25% Triton X-100 (Sigma-Aldrich) for 10 minutes and further blocking with normal goat serum blocking buffer (ZSGB-BIO) for 30 minutes at room temperature. Afterward, the cells were incubated with primary antibody overnight at 4°C. Then, the cells were washed 3 times with PBS and incubated with a secondary antibody for 1 hour, followed by Hoechst 33342 (Invitrogen, Thermo Fisher Scientific) for 2 minutes. After that, the cells were washed 3 times and mounted with an antifade mounting medium (Beyotime Biotechnology). Images were captured using a confocal microscope (Zeiss LSM880 with Airyscan).

### Cell viability and proliferation assay.

Cells were seeded in 96-well plates at a density of 2,000 cells/well. Cell viability was measured using MTT assays each day for 4 days. The results are presented as the mean ± SD of 3 independent experiments.

### Boyden chamber assays.

Cell migration and invasion were examined using 24-well Boyden chambers (Becton, Dickinson, and Co.) with 8 μm inserts coated with (invasion) or without (migration) Matrigel (Becton, Dickinson, and Co.), as previously described. A total of 0.5 × 10^5^ (143B, U2OS) or 1 × 10^5^ (U2OS/MTX300) cells per well in 200 μL of serum-free DMEM were plated on the inserts and cultured at 37°C in the upper chambers for 8 hours and 24 hours, respectively. Cells that crossed the inserts were fixed with 4% paraformaldehyde and stained with crystal violet (0.005%; Sigma-Aldrich). The images were taken using phase-contrast microscopy (Nikon), and the cell numbers were counted using ImageJ.

### Immunoblotting and IP.

For Western blot analysis, the cells were washed with cold PBS and then lysed in RIPA buffer (50 mM Tris-HCl, pH 8.0, 150 mM NaCl, 5 mM EDTA, 0.5% NP-40) containing protease inhibitor and phosphatase inhibitor cocktails (Thermo Fisher Scientific). Lysates were cleared by centrifugation at 15,294*g* for 20 minutes at 4°C. The supernatants were incubated with antibody beads or agarose overnight at 4°C, or antibodies overnight at 4°C, followed by incubation with protein A/G PLUS-Agarose (Santa Cruz Biotechnology) at 4°C for 2 hours. The beads were then washed 5 times with cold RIPA buffer and eluted with 5X SDS loading buffer. The immunoprecipitates were separated by SDS-PAGE and transferred to a PVDF membrane (MilliporeSigma, Merck). The membranes were blocked in TBS with 5% nonfat milk and 0.1% Tween20 for 1 hour at room temperature and probed with primary antibodies overnight at 4°C, washed 5 times by TBS containing 0.1% Tween20, and then incubated with secondary horseradish-peroxidase–conjugated antibodies (Promega). Clarity Western ECL substrate (Bio-Rad) was used for detection. The antibodies we used are listed in the Supplemental Table 7.

### Nuclear and cytoplasmic extraction.

Nuclear and cytoplasmic extraction were performed according to the instructions of the NE-PER Nuclear and Cytoplasmic Extraction Kit (Thermo Fisher Scientific, 78833).

### ER extraction.

ER was extracted using an ER isolation kit (Sigma-Aldrich, Merck, ER0100) following the manufacturer’s instruction. Briefly, 1 × 10^8^ 143B or U2OS/MTX300 cells were collected and centrifuged at 600*g* for 5 minutes. After being washed in ice-cold PBS, cells were resuspended in 3 mL of 1X hypotonic extraction buffer and incubated on ice for 20 minutes to allow cell swelling. Swollen cells were centrifuged at 600*g* for 5 minutes at 4°C, and the resulting cell pellets were homogenized in 2 mL of 1X isotonic extraction buffer and sequentially centrifuged at 1,000*g* for 10 minutes at 4°C to remove cell debris and nuclear material, followed by centrifugation at 12,000*g* for 15 minutes at 4°C to remove mitochondria. The supernatant was collected and ultracentrifuged at 100,000*g* for 1 hour to isolate the ER fraction. The supernatant was discarded, and the ER pellet at the bottom of the tube was resuspended in 0.4 mL of 1X isotonic extraction buffer and subjected to further analysis.

### In vitro kinase assay.

HEK293T cells overexpressing SFB-tagged MLF2 or V5-tagged PIM3 were lysed in RIPA buffer (50 mM Tris-HCl, pH 8.0, 150 mM NaCl, 5 mM EDTA, 0.5% NP-40) containing protease inhibitor and phosphatase inhibitor cocktails (Thermo Fisher Scientific). After centrifugation, the supernatants were incubated with streptavidin-conjugated beads (Amersham) or V5-conjugated beads (Thermo Fisher Scientific) overnight at 4°C. The beads were washed 5 times with RIPA buffer and then eluted with RIPA buffer containing 2 mg/mL biotin (MilliporeSigma, Merck) or V5 peptide for 4 hours. The elutes containing MLF2-SFB and V5-PIM3 were incubated with kinase buffer (Cell Signaling Technology) and 200 nM ATP (Cell Signaling Technology) for 1 hour at 37°C and then subjected to SDS-PAGE and Western blotting.

### CRISPR genome editing.

The MLF2 S65A knock-in cells were generated as previously described (35). An efficient MLF2-targeting sgRNA was selected, and a DNA donor template containing mutations was designed. The annealed guide RNA oligonucleotides were inserted into a PX459 vector. The donor template was cloned into a PUC19 vector. To increase the efficiency of positive clone selection, a fragment encoding EGFP was inserted into the donor template in the intron sequence between exon 4 and exon 5. Additionally, an internal ribosome entry site was placed upstream of EGFP. The generated donor constructs and sgRNAs were then cotransfected into 143B cells. Twenty-four hours after transfection, the cells were selected with puromycin at 0.5 μg/mL for another 48 hours. EGFP-positive cells were sorted via flow cytometry and seeded into 96-well plates to obtain single clones. Site-specific PCR and Sanger sequencing were used to validate the gene-edited clone. The sequences of the S65A knock-in donor template and sgRNA are provided in the Supplemental Table 6.

### Xenograft experiments.

Male athymic BALB/C nude mice (4–6 weeks old) were purchased from Beijing Vital River Laboratory Animal Technology. For the orthotopic osteosarcoma metastasis model, 1.2 × 10^6^ U2OS/MTX300 cells and 7.5 × 10^5^ 143B cells stably expressing luciferase were transplanted orthotopically into the bones of male 4- to 6-week-old BALB/c nude mice, as previously described (54, 55). Lung metastasis in these mice was monitored using the IVIS Lumina System (PerkinElmer) began 2 weeks after injection. The mice were sacrificed when a significant difference in metastasis was observed between the treatment groups and the tumor size was less than 1.5 cm in diameter. The lungs then were harvested for H&E staining.

### Statistics.

GraphPad Prism (version 9.4.1) software was used for statistical analysis. The data are presented as the mean ± SD. The error bars indicate the SD. Two-tailed Student’s *t* tests and 2-way ANOVA were used to compare the differences between groups. The correlation coefficients and *P* values were assessed using nonparametric Spearman’s tests. Differences were considered significant when *P* values were less than 0.05.

### Study approval.

The use of human osteosarcoma tissues was reviewed and approved by the Ethics Committee of SYSUCC (approval B2022-620-01). The animal experiments were approved by the Animal Research Committee of SYSUCC (approval 20100F) and were performed in strict accordance with the US NIH’s *Guidelines for the Care and Use of Laboratory Animals* and the *US Government Principles for the Utilization and Care of Vertebrate Animals Used in Testing, Research, and Training*.

### Data availability.

The in vivo CRISPR screening data and ProSRSA data generated in this study have been deposited in the Sequence Read Archive (SRA) database under BioProject accession PRJNA1189506, and RNA-Seq data have been deposited in SRA database under BioProject accession PRJNA1188347. The MS raw data are included in Supplemental Table 2 for STUB1and Supplemental Table 4 for MLF2. Supporting data values can be found in the Supporting Data Values file. All data supporting the findings of this study are available within the main text and supplemental materials.

## Author contributions

TK, D Liao, LZ, and CZ conceived and designed the project. CZ performed most of the experiments and analyzed the data. XW collected clinical samples and provided critical discussions. JZ, YZ, JD, WL, WC, XY, and D Lin provided experimental support. RZ provided technical assistance. SW supported the CRISPR genome editing. JL and QZ analyzed the CRISPR screening data. TK, D Liao, LZ, and CZ wrote the manuscript, and the other authors helped revise and proofread the manuscript. All coauthors have seen and approved the manuscript.

## Supplementary Material

Supplemental data

Unedited blot and gel images

Supplemental table 1

Supplemental table 2

Supplemental table 3

Supplemental table 4

Supplemental table 5

Supplemental table 6

Supplemental table 7

Supporting data values

## Version 1 10/15/2025

Electronic publication
