# Expression of Concern for PIM3-mediated phosphorylation stabilizes myeloid leukemia factor 2 to promote metastasis in osteosarcoma

**DOI:** 10.1172/JCI213311

**Published:** 2026-10-01

**Authors:** Cuiling Zeng, Xin Wang, Jinkun Zhong, Yu Zhang, Ju Deng, Wenqiang Liu, Weixuan Chen, Xinhao Yu, Dian Lin, Ruhua Zhang, Shang Wang, Jianpei Lao, Qi Zhao, Li Zhong, Tiebang Kang, Dan Liao

Original citation: *J Clin Invest.* 2025;135(20):e191040. https://doi.org/10.1172/JCI191040

Citation for this expression of concern: *J Clin Invest.* 2026;136(19):e213311. https://doi.org/10.1172/JCI213311

The authors recently alerted the Editors to errors in the source data for Figure 1G and Supplemental Figure 10K as well as errors in the statistical analysis of Figure 2, Q–T and Figure 5R that were identified in response to an institutional mandate for a post-publication audit of the data. Although the authors have indicated that the errors were due to inadvertent mistakes, the Editors are issuing this Expression of Concern to alert readers to the issues. We have requested institutional oversight into this matter and will alert readers to the outcome when the institutional evaluation is complete.

## Version 1 10/01/2026

Electronic publication

