# Supplementary material for: PIM3-mediated phosphorylation stabilizes myeloid leukemia factor 2 to promote metastasis in osteosarcoma
Source: J Clin Invest. 2025 Oct 15;135(20):e191040. doi: 10.1172/JCI191040 (PMC12520695; doi:10.1172/JCI191040)
Supplement: Supplemental data [file jci-135-191040-s031.pdf]

Supplementary Information

PIM3-mediated phosphorylation stabilizes myeloid leukemia factor 2 to promote metastasis in osteosarcoma

Cuiling Zeng,[1] Xin Wang,[1] Jinkun Zhong,[1] Yu Zhang,[2] Ju Deng,[1] Wenqiang Liu,[1] Weixuan chen,[1] Xinhao Yu,[1] Dian Lin,[1] Ruhua Zhang,[1] Shang Wang,[3] Jianpei Lao,[1] Qi Zhao,[1] Li Zhong,[4] Tiebang Kang,[1] and Dan Liao[1]

Supplemental Tables 1 CRISPRa screening results and raw read counts of the input and lung tissues

Supplemental Tables 2 STUB1 interacting proteins identified by tandem affinity purification and mass spectrometry

Supplemental Tables 3 RNA-seq analysis result of U2OS cells expressing Vector or MLF2 (MLF2 vs VEC)

Supplemental Tables 4 MLF2 interacting proteins identified by tandem affinity purification and mass spectrometry

Supplemental Tables 5 Result of Pro-SRSA generated by MAGeCK (MLF2 high vs MLF2 low)

Supplemental Tables 6 Oligonucleotides sequences summary

Supplemental Tables 7 Antibodies and Reagents

Supplemental Figures

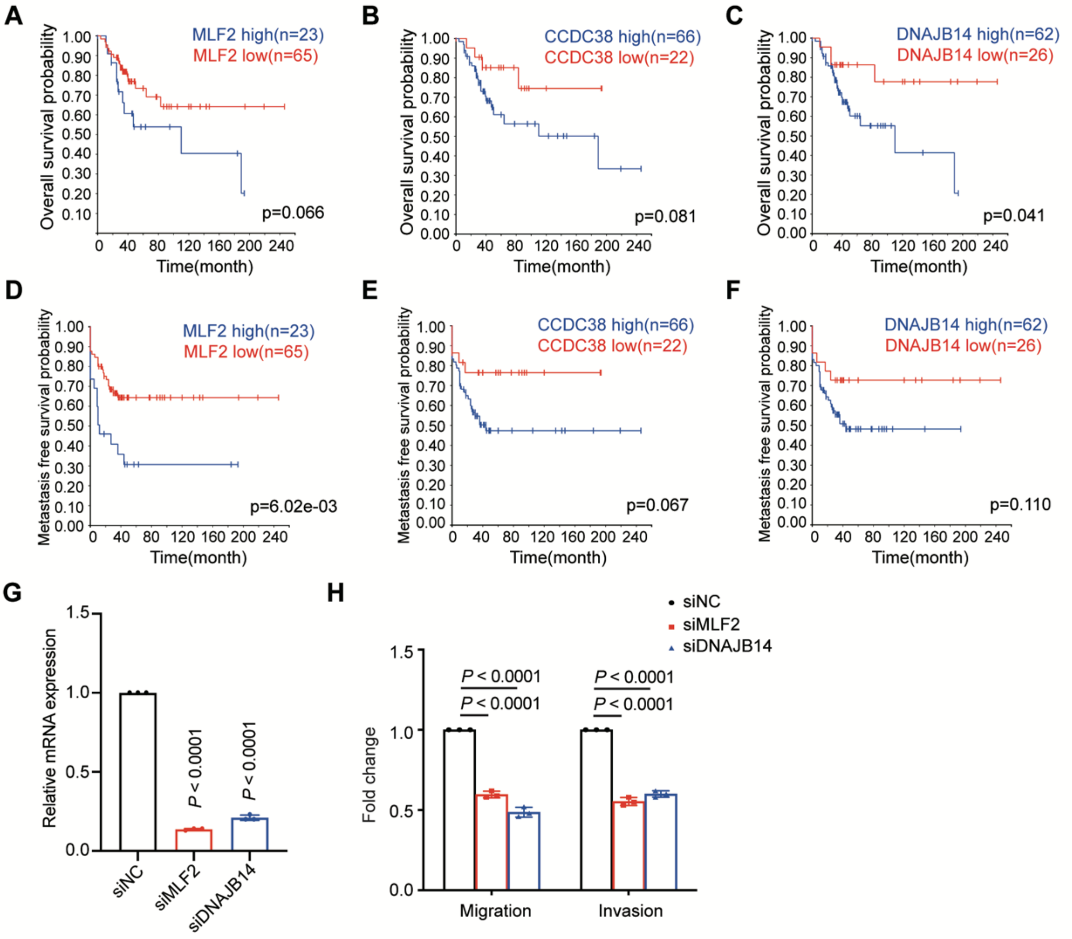

Supplemental Figure 1. In vivo genome-wide CRISPR activation screening identifies genes promoting lung metastasis of osteosarcoma. (A-F) The clinical relevance of the indicated genes in osteosarcoma was determined using the Mixed Osteosarcoma (Mesenchymal) - Kuijjer - 127 - vst - ilmnhwg6v2 dataset from the R2 database (R2: Genomics Analysis and Visualization Platform). Patients were divided into two classes using top quartile value (MLF2) or lower quartile value (CCDC38 and DNAJB14) as cutoff based on their expression distributions. **(G** and **H)** U2OS/MTX300 cells transfected with MLF2 or DNAJB14 targeted siRNAs for 24 hours were subjected to qPCR **(G)** and transwell assays **(H)**. *n* = 3 biologically independent experiments. Data are presented as mean ± SD. *P* values were calculated using 1-way ANOVA with Dunnett's test.

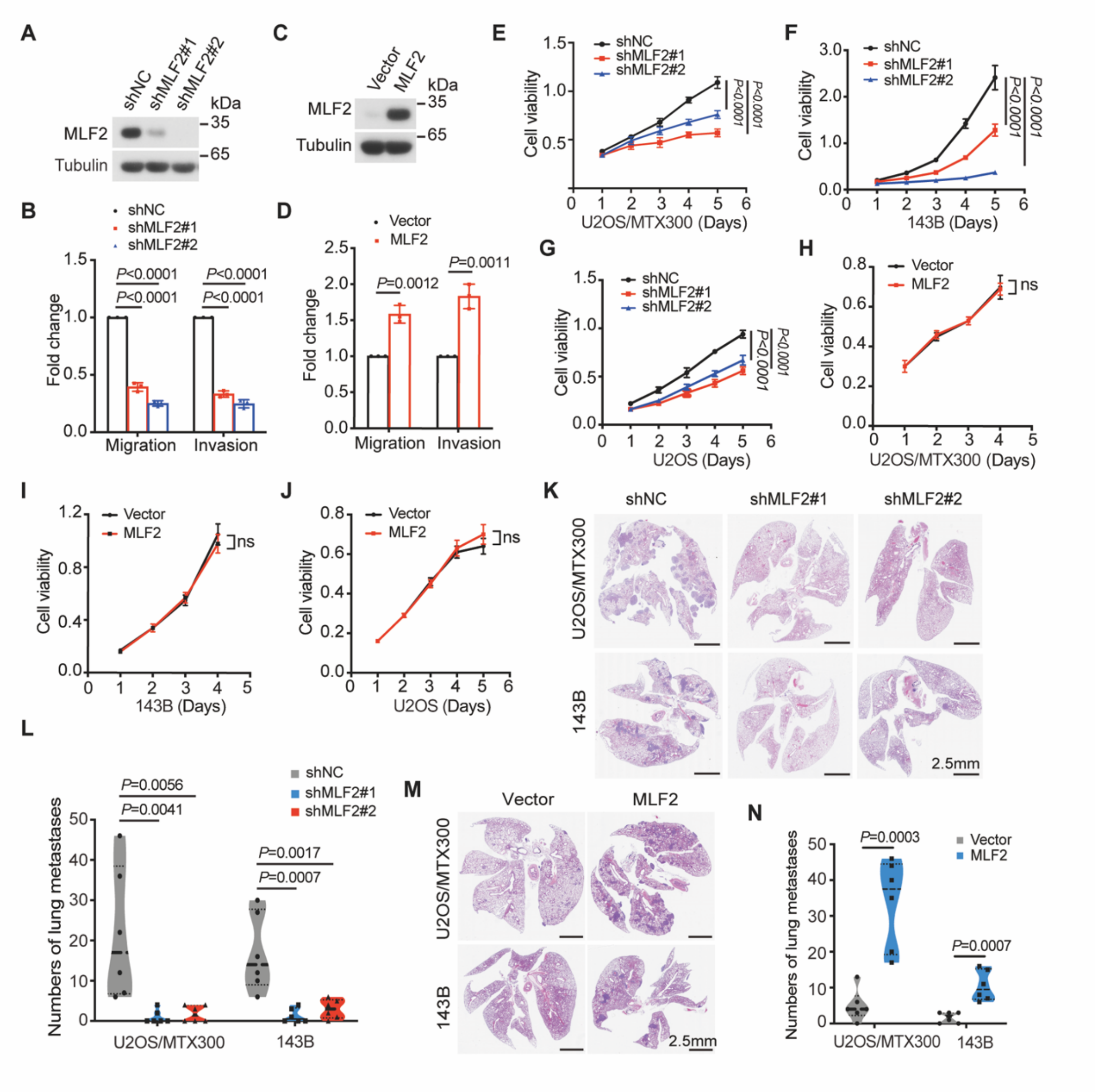

Supplemental Figure 2. MLF2 promotes lung metastasis in osteosarcoma. (A and C) The expression level of MLF2 were analyzed by Western blotting in the indicated U2OS cells stably expressing MLF2-targeted shRNAs or overexpression of MLF2. Data are representative of *n* = 3 independent experiments. **(B and D)** Quantification analyses of migration and invasion assays using U2OS cells stably expressing MLF2-targeted shRNAs or overexpression of MLF2. *n* = 3 biologically independent experiments. **(E-J)** Cell viability was analyzed by MTT assay in indicated U2OS/MTX300, 143B and U2OS cells stably expressing MLF2-targeted shRNAs or overexpression of MLF2. Data are representative of *n* = 3 independent experiments. **(K and M)** Haematoxylin and eosin (H&E) staining of lungs of representative mice orthotopically injected with the indicated stable cells. Scale bars, 2.5 mm. **(L and N)** Quantification analyses of lung nodules in **K** and **M**. *n* = 6 mice per group. Data in **B-J, L** and **N** are presented as mean ± SD. *P* values were calculated using 1-way ANOVA with Dunnett's test (**B** and **L**), Two-way ANOVA test (**E-J**), and two-tailed Student's t-test (**D** and **N**).

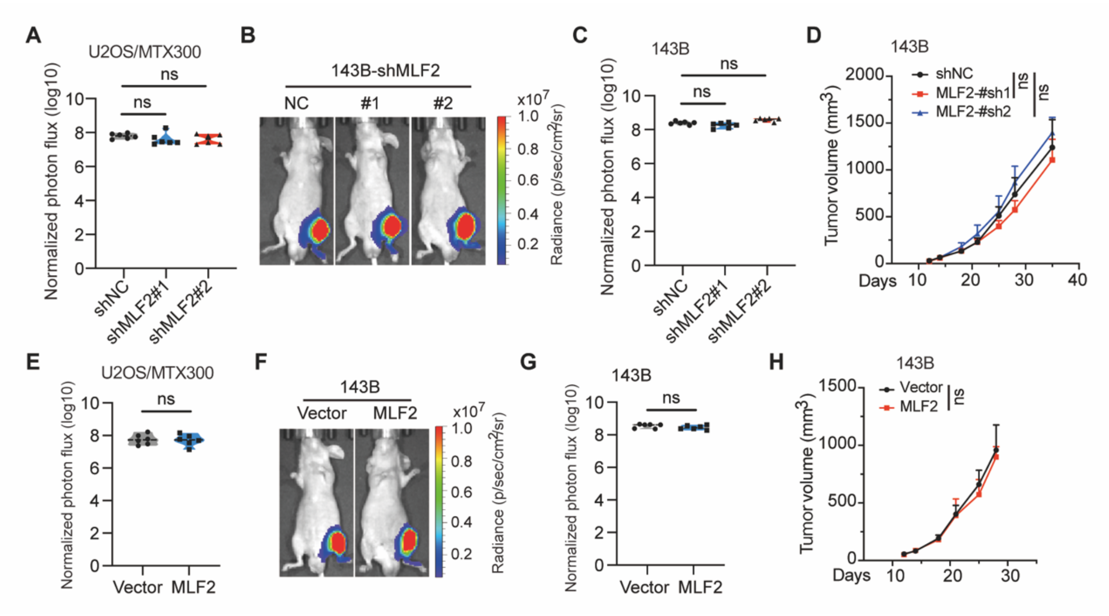

Supplemental Figure 3. Alteration of MLF2 does not affect primary tumor growth in osteosarcoma. (A and E) Quantification analyses of the bioluminescence images of mice orthotopically transplanted with the indicated luciferase-transduced U2OS/MTX300 cells. **(B-D and F-H)** The indicated luciferase-transduced 143B cells stably expressing MLF2-targeted shRNAs or overexpression of MLF2 were orthotopically injected into mice. Representative bioluminescent images of mice at the end point **(B and F)**. Quantification analyses of the bioluminescence images **(C and G)**. Tumor growth was measured at the indicated time points **(D and H)**. n = 6 mice per group. Data in **A, C-E, G** and **H** are presented as mean ± SD. *P* values were calculated using 1-way ANOVA with Dunnett's test (**A**, **C** and **D**,) and two-tailed Student's t-test (**E**, **G** and **H**), ns means no significance.

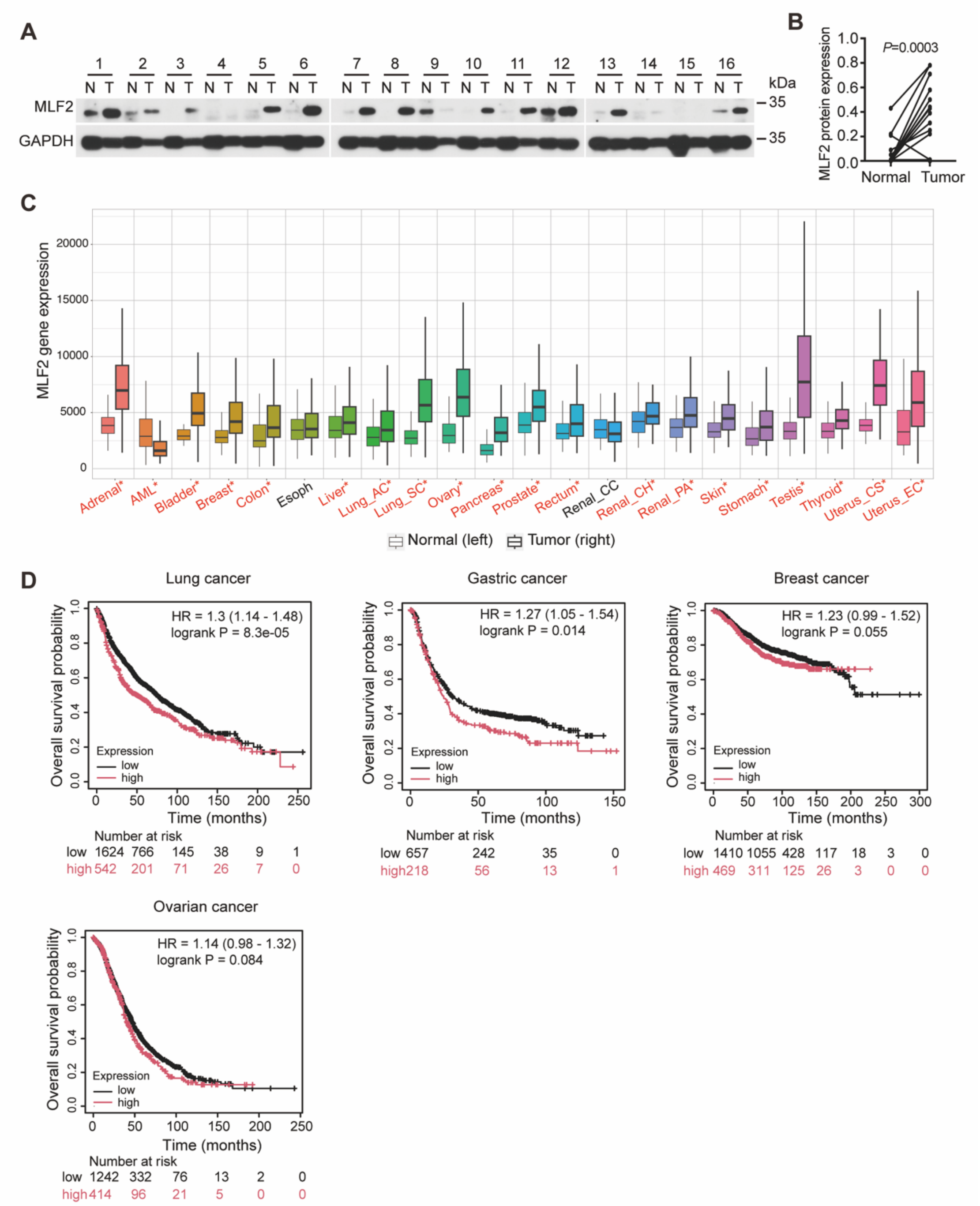

Supplemental Figure 4. High expression of MLF2 is associated with poor prognosis in cancer. (A and **B)** Western blotting analysis and quantification of MLF2 protein levels in human osteosarcoma tissues. T: tumor, N: normal. *P* values were calculated using two-tailed Student's t-test**. (C)** The mRNA expression levels of MLF2 in pan-cancer were analyzed using RNA Seq data via TNMplot. Box plots display expression distributions with horizontal lines indicating median values for each group. Red*: Mann-Whitney p<0.05 and expression >10 in tumor or normal. **(D)** The clinical relevance of MLF2 in other cancers were determined using the publicly accessible online tool KM Plotter. Patients were divided into two classes using top quartile value as

cutoff based on their expression distributions. Gene chip data were selected for the correlations analysis.

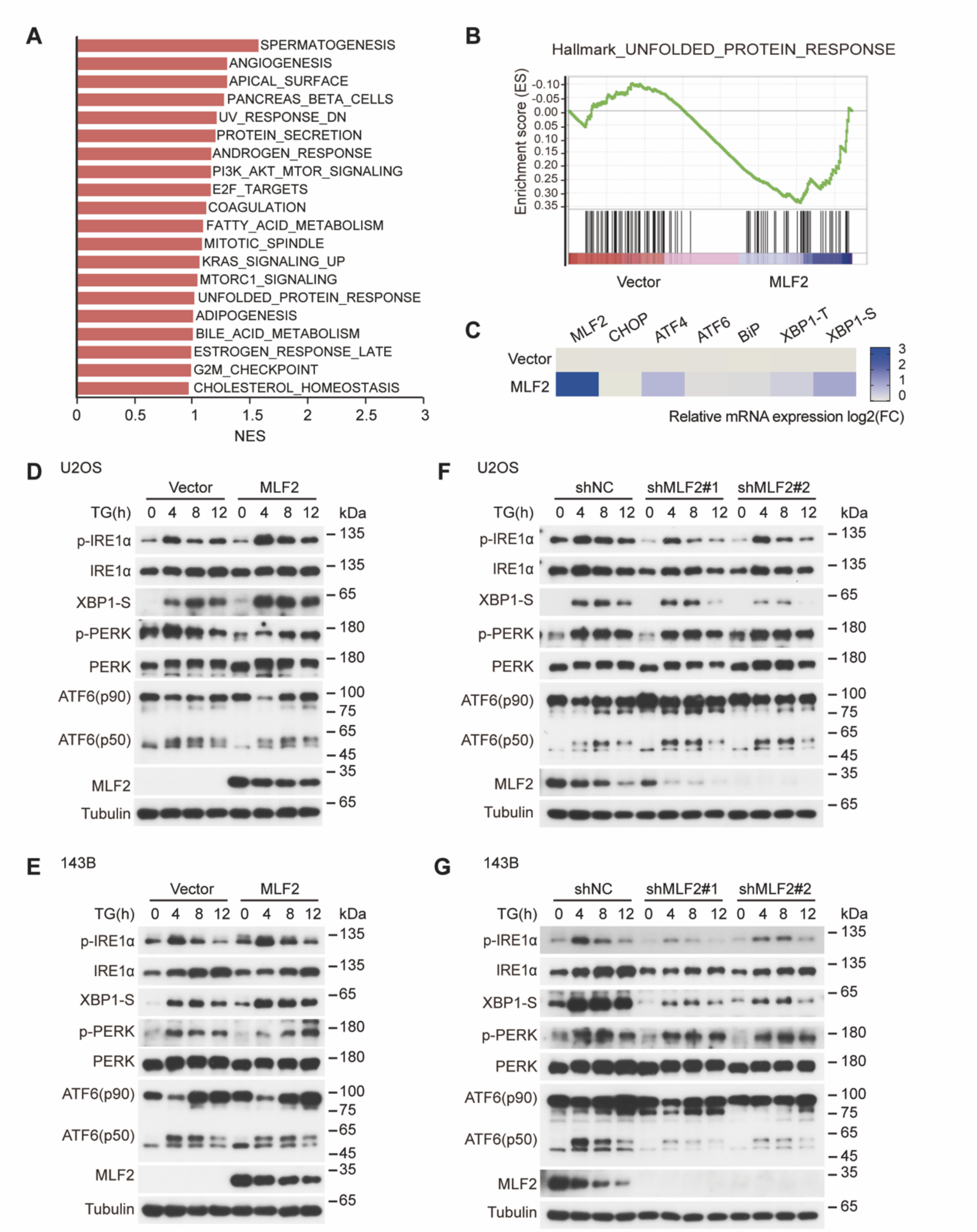

Supplemental Figure 5. MLF2 activates IRE1α/XBP1-S signaling in osteosarcoma cells. (A) Barplots showing the enriched pathways by NES in U2OS cells with MLF2 overexpression. **(B)** Hallmark pathway UNFOLDED PROTEIN RESPONSE enriched by GSEA on combined data of Vector and MLF2 overexpression in U2OS cells. **(C)** The relative mRNA levels of ER stress markers were normalized to the GAPDH level in U2OS MLF2 overexpression stable cells by qPCR. **(D** and **E)** The indicated proteins were analyzed by Western blotting in the indicated U2OS and 143B cells stably overexpression of MLF2 and treated with 1μM

thapsigargin (TG) for the indicated time. The experiments were repeated three times independently with similar results. **(F** and **G)** The indicated proteins were analyzed by Western blotting in the indicated U2OS and 143B cells stably expressing MLF2-targeted shRNAs and treated with 1μM thapsigargin (TG) for the indicated time. The experiments were repeated three times independently with similar results.

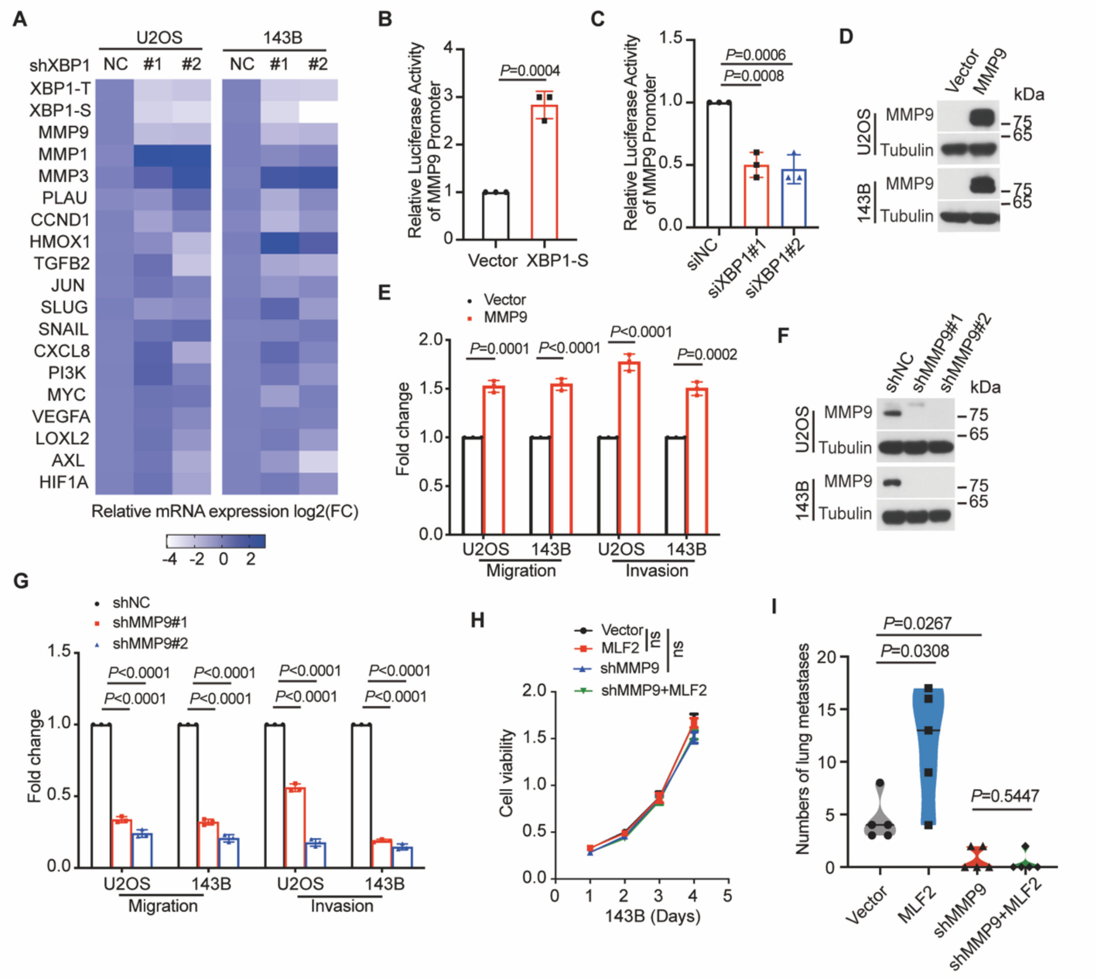

Supplemental Figure 6. XBP1-S/MMP9 axis promotes cell migration and invasion in osteosarcoma cells. (A) Heat maps of differential expression gene in the indicated U2OS and 143B MLF2 knockdown stable cells from quantitative real-time PCR analysis. **(B** and **C)** U2OS cells transfected with XBP1-S or XBP1-targeted siRNAs for 24 hours were transfected with the MMP9-Luc reporter for another 24 hours and then subjected to the luciferase activity assay as described in Methods section. **(D** and **F)** The expression level of MMP9 were analyzed by Western blotting in the indicated stable cells. Data are representative of n = 3 independent experiments. **(E** and **G)** Quantification analyses of migration and invasion assays using the indicated U2OS and 143B cells stably overexpression of MMP9 or expressing MMP9-targeted shRNAs. **(H)** Cell viability was analyzed by MTT assay in the indicated 143B stable cells. Data are presented as mean ± SD and are representative of n = 3 independent experiments. Two-way ANOVA test was performed, and ns means no significance. **(I)** Quantification analyses of lung nodules from mice orthotopically injected with the indicated stable cells. n = 5 mice per group. Data are means ± SD. P values were calculated using two-tailed Student's t-test followed by Benjamini-Hochberg correction. Data in **B, C, E** and **G** are

presented as mean ± SD. *n* = 3 biologically independent experiments. *P* values were calculated using two-tailed Student's t-test (**B** and **E**) and 1-way ANOVA with Dunnett's test (**C** and **G**).

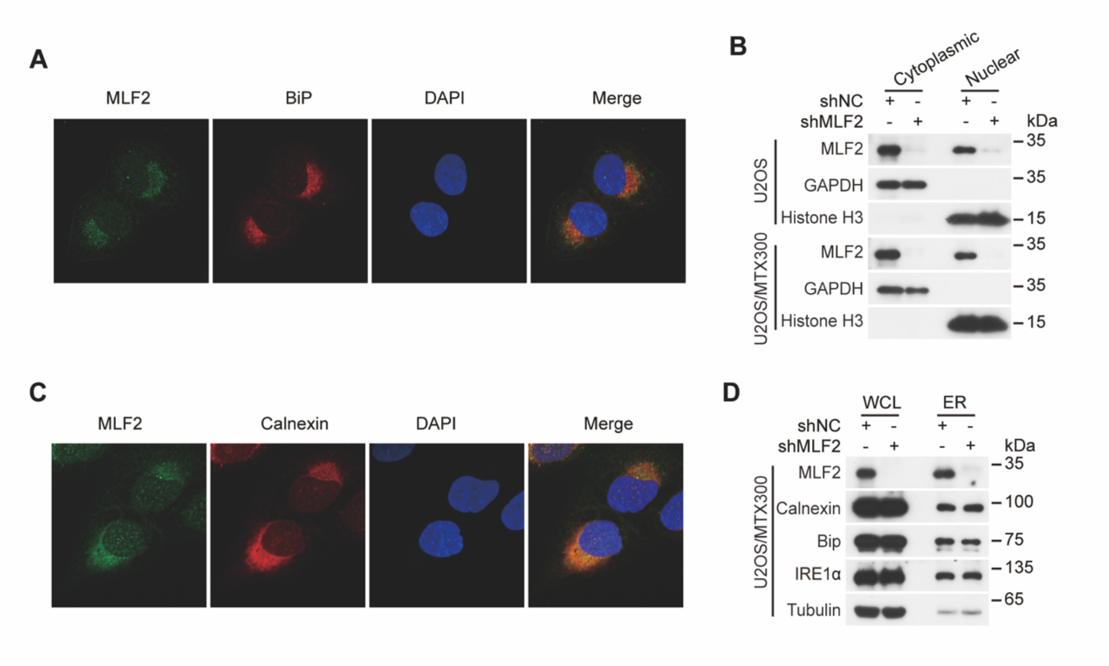

Supplemental Figure 7. MLF2 was co-located with BiP in ER.

(A and **C)** Representative immunofluorescence pictures of U2OS cells. ER was labeled with Calnexin (red), MLF2 (green) and BiP (red) were labeled with antibody and DNA was visualized with DAPI (blue). **(B)** The nuclear and cytoplasmic location of MLF2 in U2OS and U2OS/MTX300 cells were analyzed by NE-PER Nuclear and Cytoplasmic Extraction Kit. **(D)** The ER location of MLF2 in U2OS/MTX300 cells was analyzed by ER enrichment Kit. Data in **B** and **D** are representative of n = 3 independent experiments.

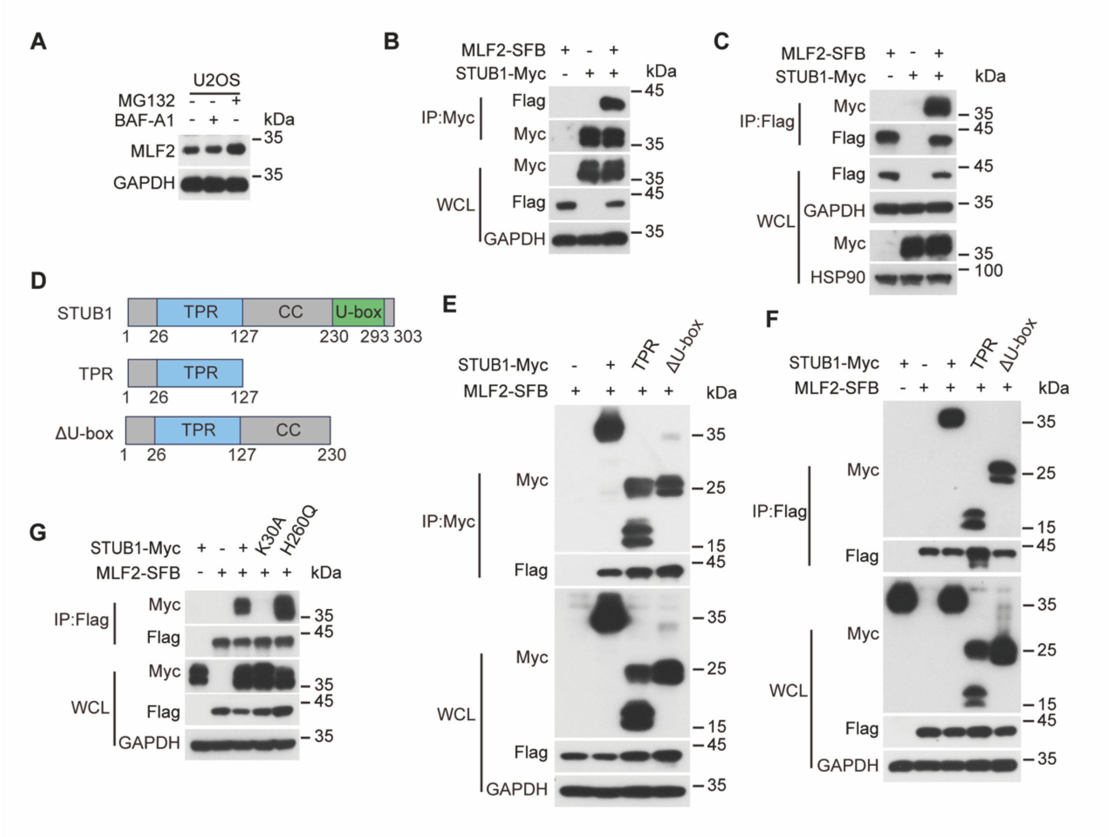

Supplemental Figure 8. STUB1 acts as an E3 ligase targeting MLF2 in osteosarcoma.

(A) U2OS cells were treated with MG132 (10μM) or Baf-A1 (200nM) for 4 hours were lysed and analyzed by Western blotting. The experiments were repeated three times independently with similar results. **(B** and **C)** HEK293T cells were co-transfected with MLF2-SFB and STUB1-Myc for 48 hours and then subjected to immunoprecipitation using anti-FLAG antibody or anti-Myc antibody followed by Western blotting. **(D)** Schematic illustration of STUB1 structure. **(E** and **F)** The domain structure of STUB1 interacts with MLF2, was measured by co-immunoprecipitation. HEK293T cells were co-transfected with MLF2-SFB and full-length STUB1- Myc or its various deletion mutants for 48 hours and then subjected to immunoprecipitation using anti-FLAG antibody or anti-Myc antibody followed by Western blotting. **(G)** HEK293T cells were co-transfected with MLF2-SFB and STUB1- Myc WT, K30A mutant or H260Q mutant for 48 hours and then subjected to immunoprecipitation using anti-FLAG antibody followed by Western blotting. Data in **B, C** and **E-G** are representative of *n* = 3 independent experiments.

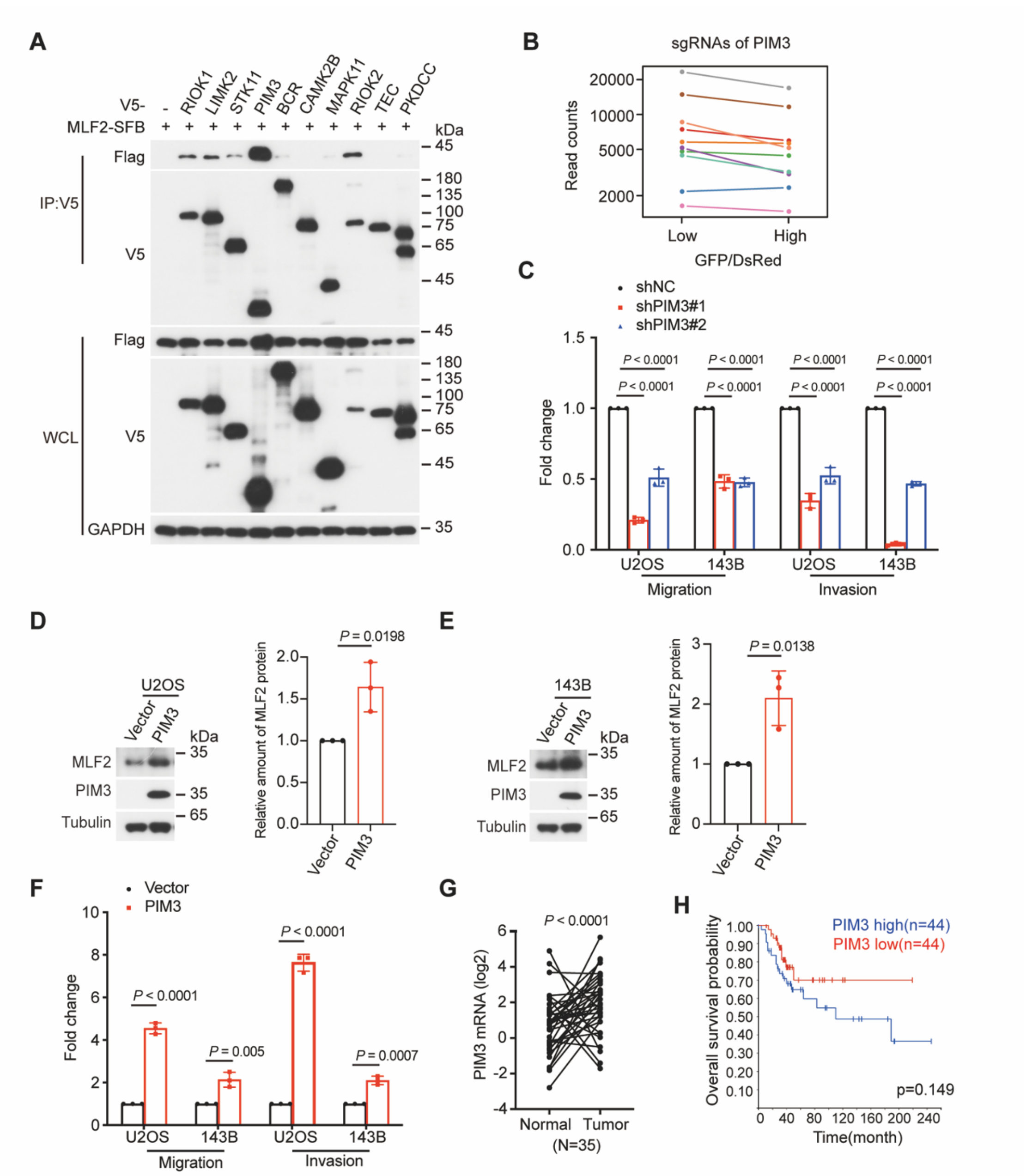

Supplemental Figure 9. PIM3 promotes the migration and invasion of osteosarcoma cells.

(A) HEK293T cells were co-transfected with MLF2-SFB and the indicated kinases for 48 hours and then subjected to immunoprecipitation using anti-V5 antibody followed by Western blotting. Data are representative of *n* = 3 independent experiments. **(B)** Read counts of individual sgRNAs targeting PIM3 were shown. **(C** and **F)** Quantification analyses of migration and invasion assays using the indicated U2OS and 143B cells stably expressing PIM3-targeted shRNAs or overexpression of PIM3. **(D** and **E)** Western blot analysis and quantification of MLF2 and PIM3 protein levels in U2OS and 143B cells with or without PIM3 overexpression. **(G)** The mRNA levels of PIM3 were analyzed by real-time PCR in paired human osteosarcoma tissues and adjacent normal tissues. **(H)** The clinical relevance of PIM3 in

osteosarcoma was determined using the Mixed Osteosarcoma (Mesenchymal) - Kuijjer - 127 - vst - ilmnhwg6v2 dataset from the R2 database (R2: Genomics Analysis and Visualization Platform). Patients were divided into two classes using the median value as cutoff based on their expression distributions. Data in **C-F** are presented as mean ± SD. *n* = 3 biologically independent experiments. *P* values were calculated using 1-way ANOVA with Dunnett's test (**C**) and two-tailed Student's t-test (**D-G**).

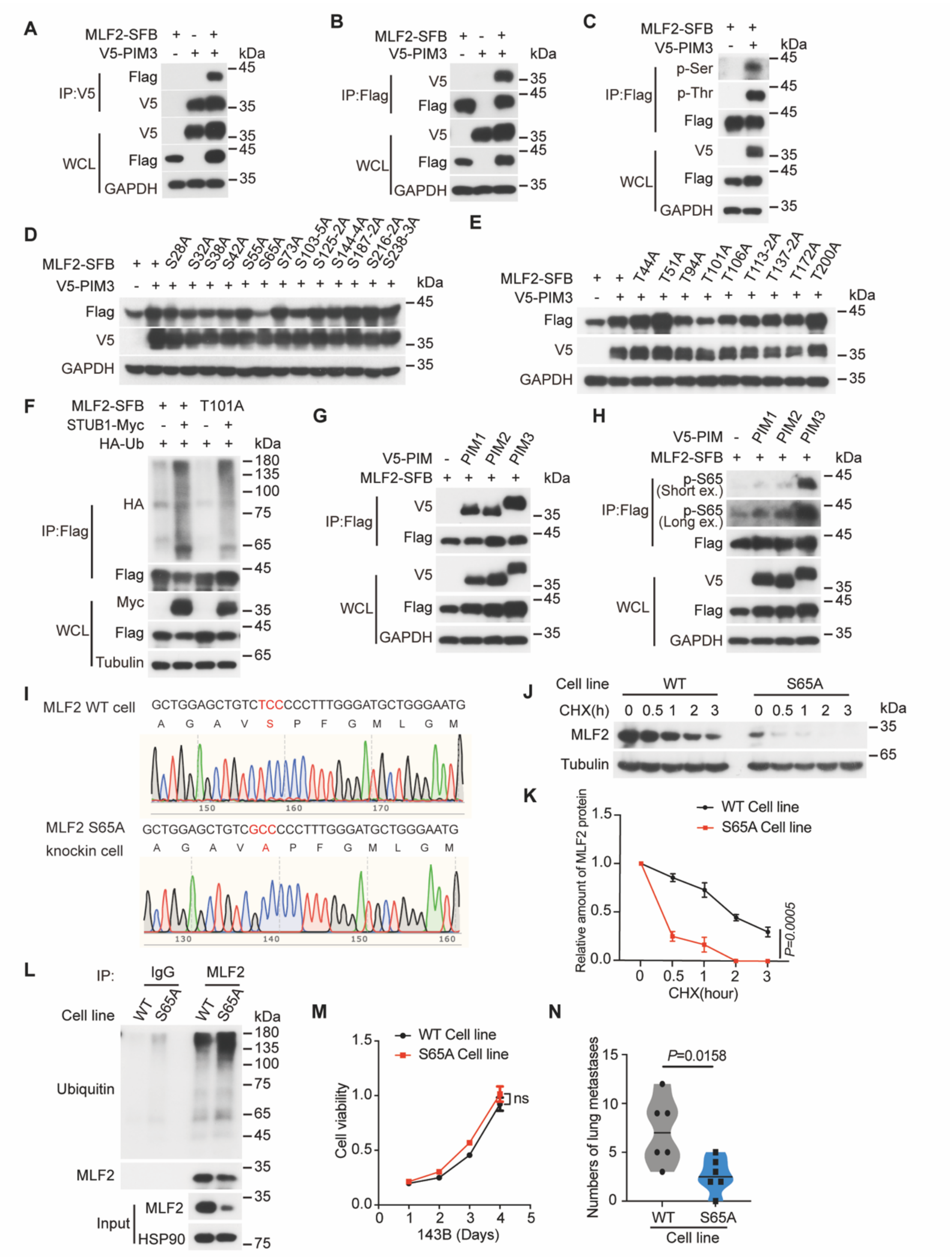

Supplemental Figure 10. PIM3 interacts with MLF2 and phosphorylate MLF2 at S65.

(A-C) HEK293T cells were co-transfected with MLF2-SFB and V5-PIM3 for 48 hours and then subjected to immunoprecipitation using anti-V5 antibody or anti-FLAG antibody followed by Western blotting. **(D** and **E)** HEK293T cells were co-transfected with the indicated plasmids for 48 hours and then subjected to Western blotting. **(F-H)** HEK293T cells were co-transfected with the indicated plasmids for 48 hours and then subjected to

immunoprecipitation using anti-FLAG antibody followed by Western blotting. **(I)** Sequencing of parental and individual clones of parental 143B cells with knock-in expression of MLF2 (S65A) mutants. **(J** and **K)** Parental MLF2 WT and S65A knock-in 143B cells were treated with 20 ug/ml cycloheximide (CHX) for the indicated times, and then were analyzed by western blot **(J)**. Quantitation of MLF2 protein levels was based on the Western blotting results **(K)**. *n* = 3 biologically independent experiments. Data are presented as mean ± SD. *P* values were calculated using two-tailed Student's t-test. **(L)** Parental MLF2 WT and S65A knock-in 143B cells were subjected to immunoprecipitation using anti-MLF2 antibody or anti-IgG antibody followed by Western blotting. **(M)** Cell viability was analyzed by MTT assay in parental MLF2 WT and S65A knock-in 143B cells. Data are presented as mean ± SD and are representative of *n* = 3 independent experiments. Two-way ANOVA test was performed, and ns means no significance. **(N)** Quantification analyses of lung nodules from mice orthotopically injected with MLF2 WT and S65A knock-in 143B cells. *n* = 6 mice per group. Data are presented as mean ± SD. *P* values were calculated using two-tailed Student's t-test. Data in **A-H**, **J** and **L** are representative of *n* = 3 independent experiments.

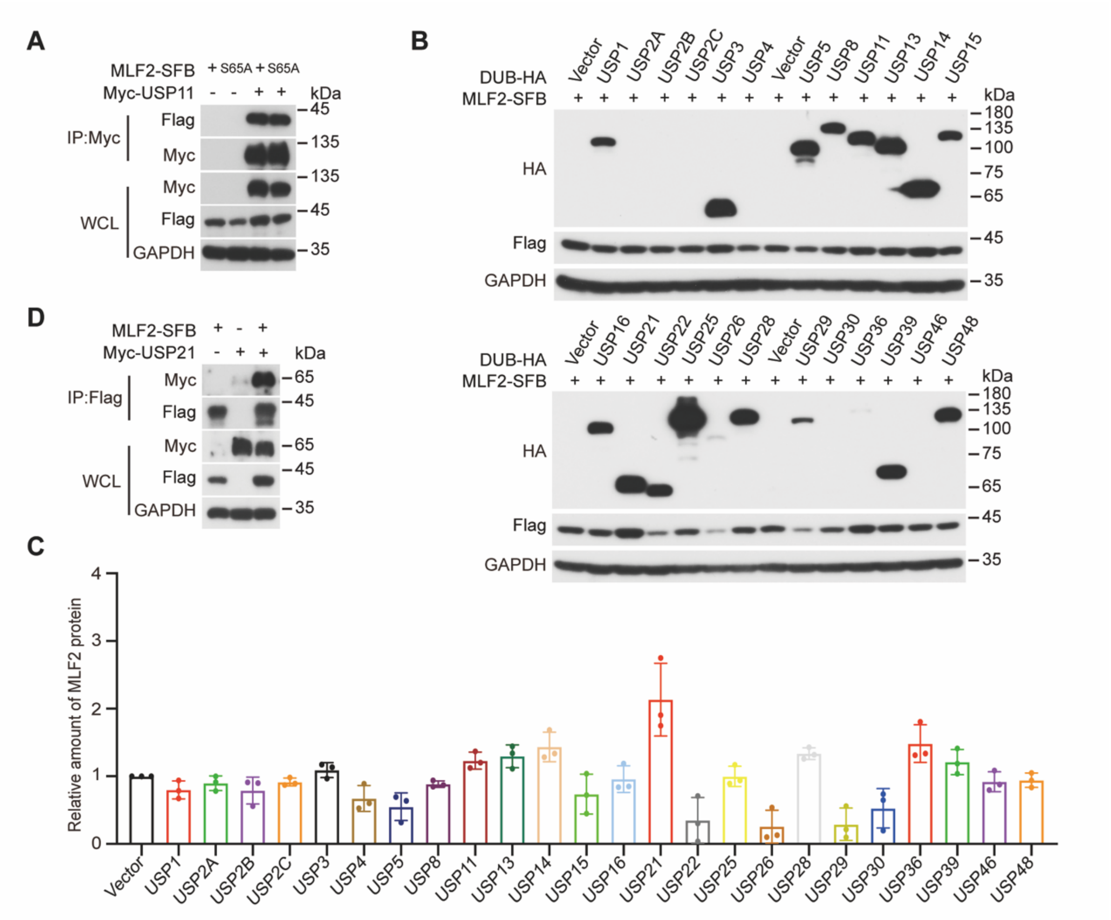

Supplemental Figure 11. USP21 interacts with and stabilizes MLF2.
(A and D) HEK293T cells were co-transfected with the indicated plasmids for 48 hours and then subjected to immunoprecipitation using anti-Myc antibody **(A)** or anti-FLAG antibody **(D)** followed by Western blotting. Data are representative of n = 3 independent experiments. **(B and C)** HEK293T cells were co-transfected with MLF2-SFB and the indicated DUBs for 48 hours and then were analyzed by Western blotting **(B)**. Quantitation of MLF2 protein levels was based on the Western blotting results **(C)**. n = 3 biologically independent experiments. Data are presented as mean ± SD. P values were analyzed using two-tailed Student's t-test.
