# Supplementary material for: PIM3-mediated phosphorylation stabilizes myeloid leukemia factor 2 to promote metastasis in osteosarcoma
Source: J Clin Invest. 2025 Oct 15;135(20):e191040. doi: 10.1172/JCI191040 (PMC12520695; doi:10.1172/JCI191040)

Full unedited blot for Figure1

Figure 1C

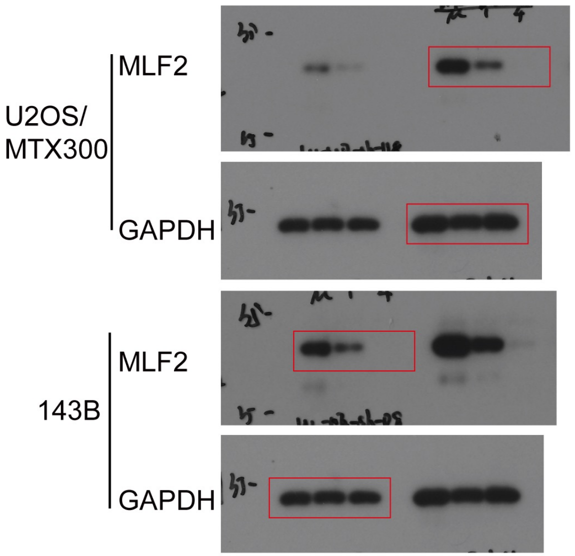

Figure 1D

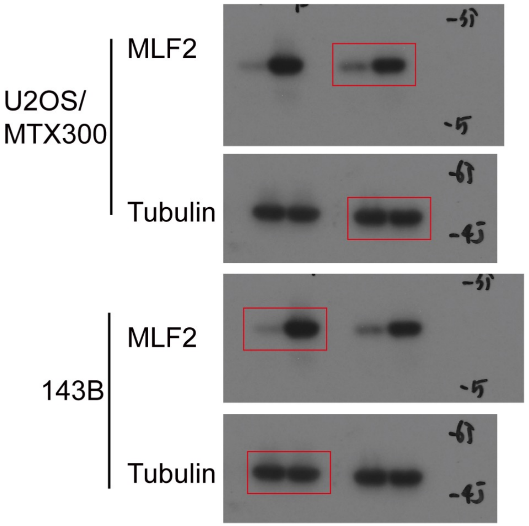

Full unedited blot for Figure2

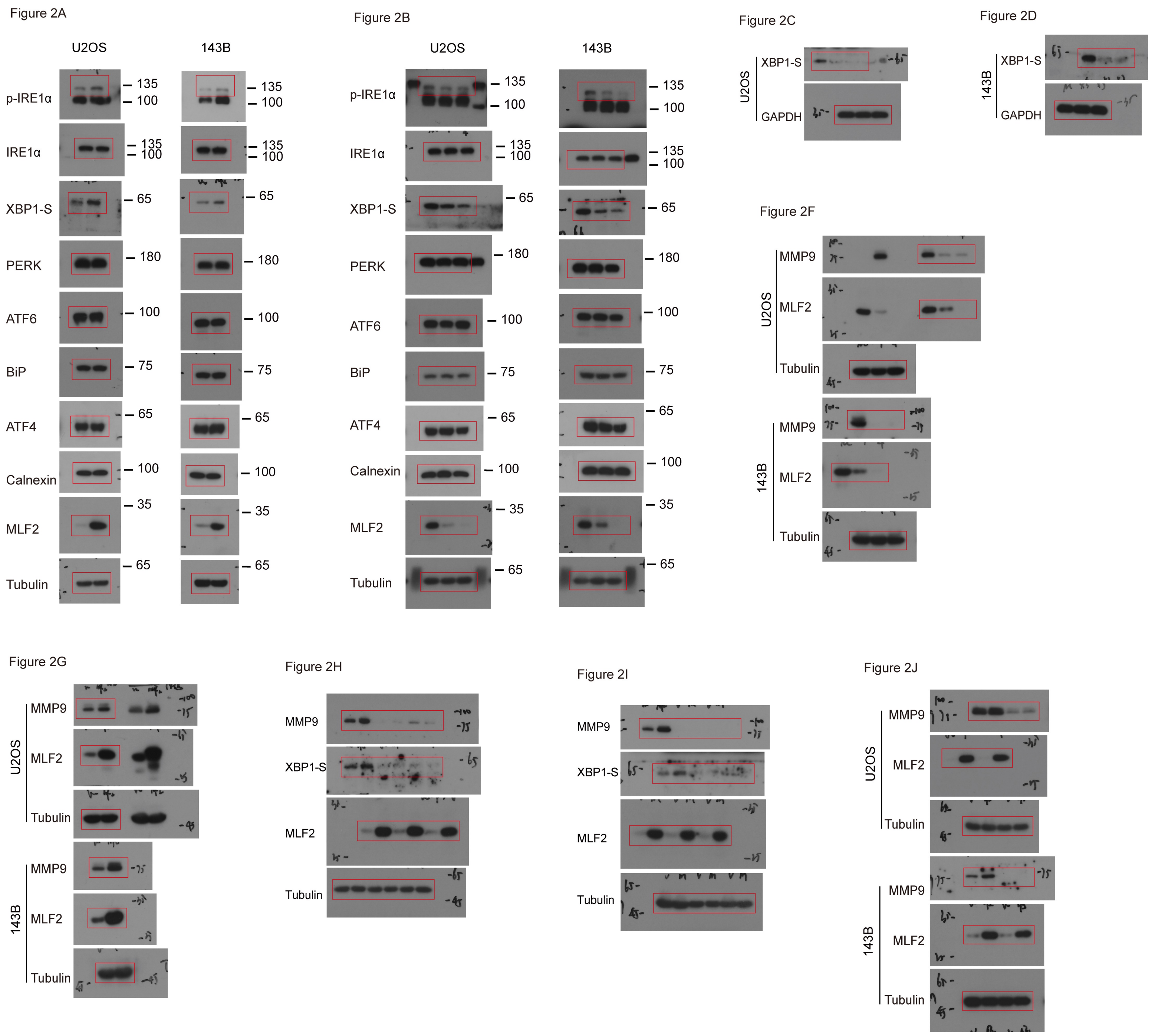

Full unedited blot for Figure3

Figure 3A

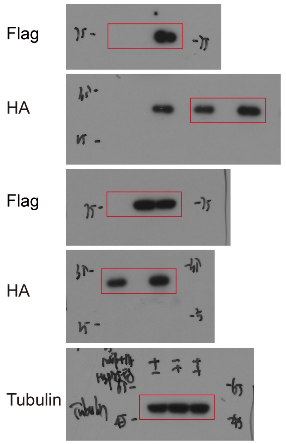

Figure 3B

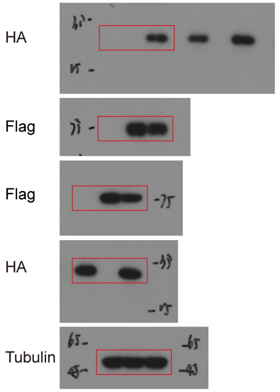

Figure 3C

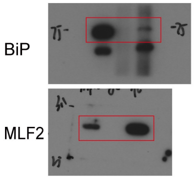

Figure 3G

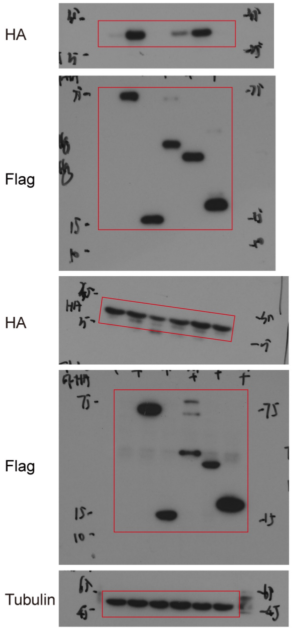

Figure 3D

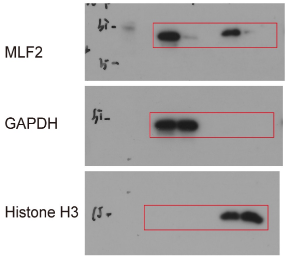

Figure 3E

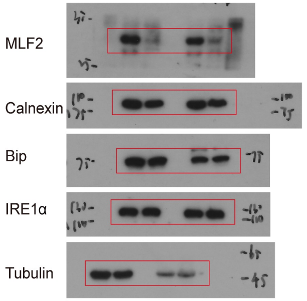

Figure 3H

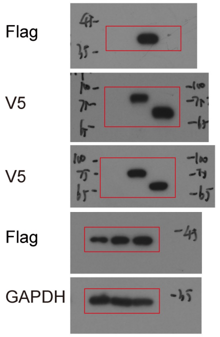

Figure 3I

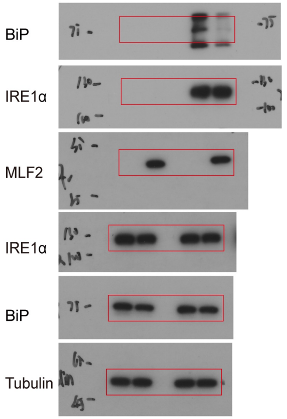

Figure 3J

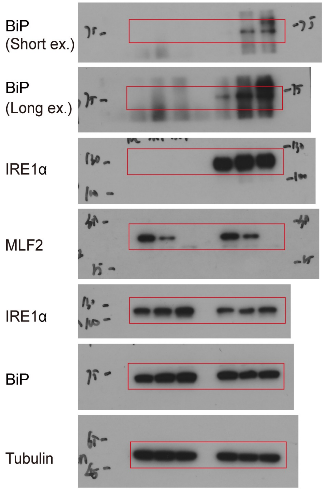

Full unedited blot for Figure4

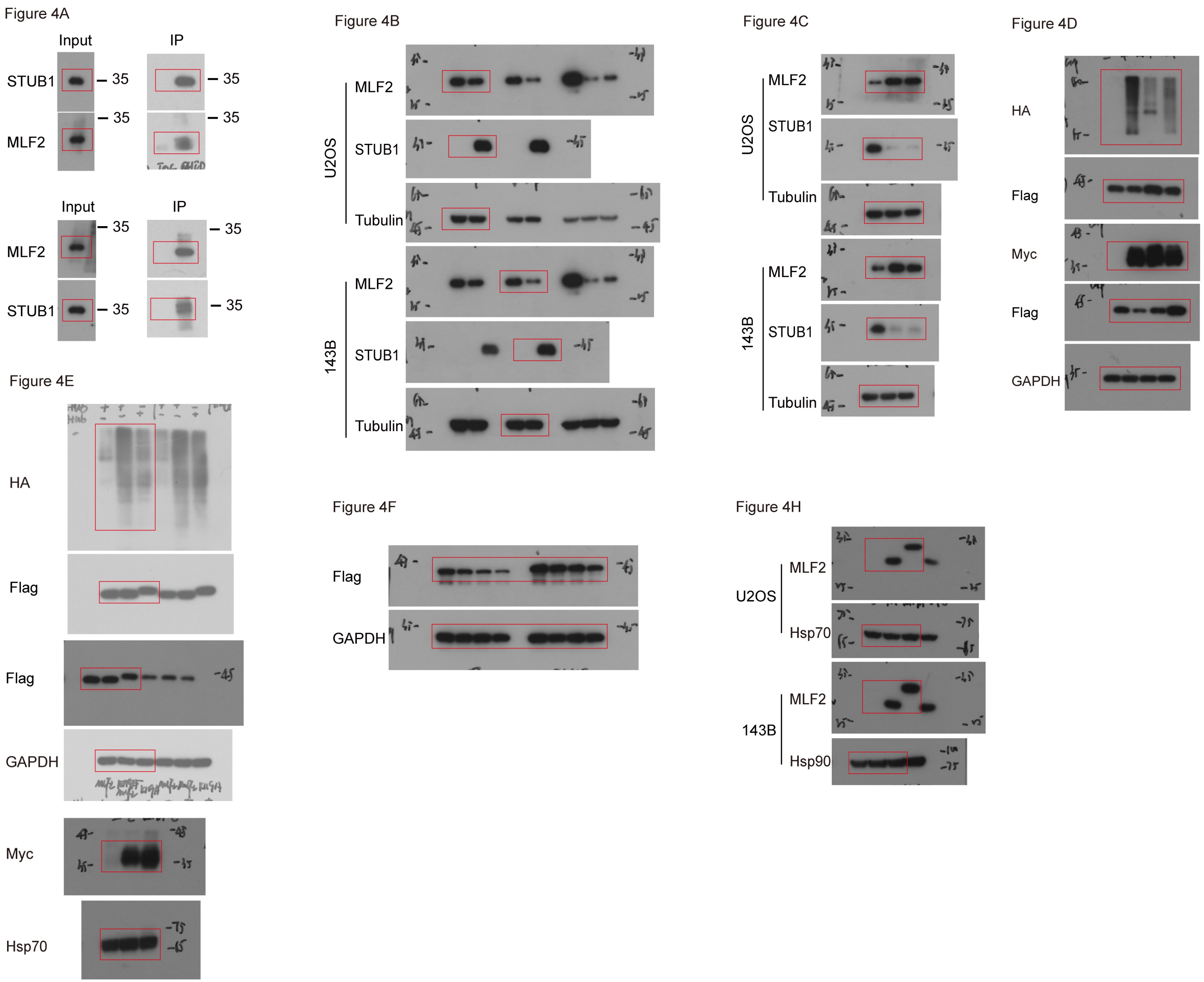

Full unedited blot for Figure5

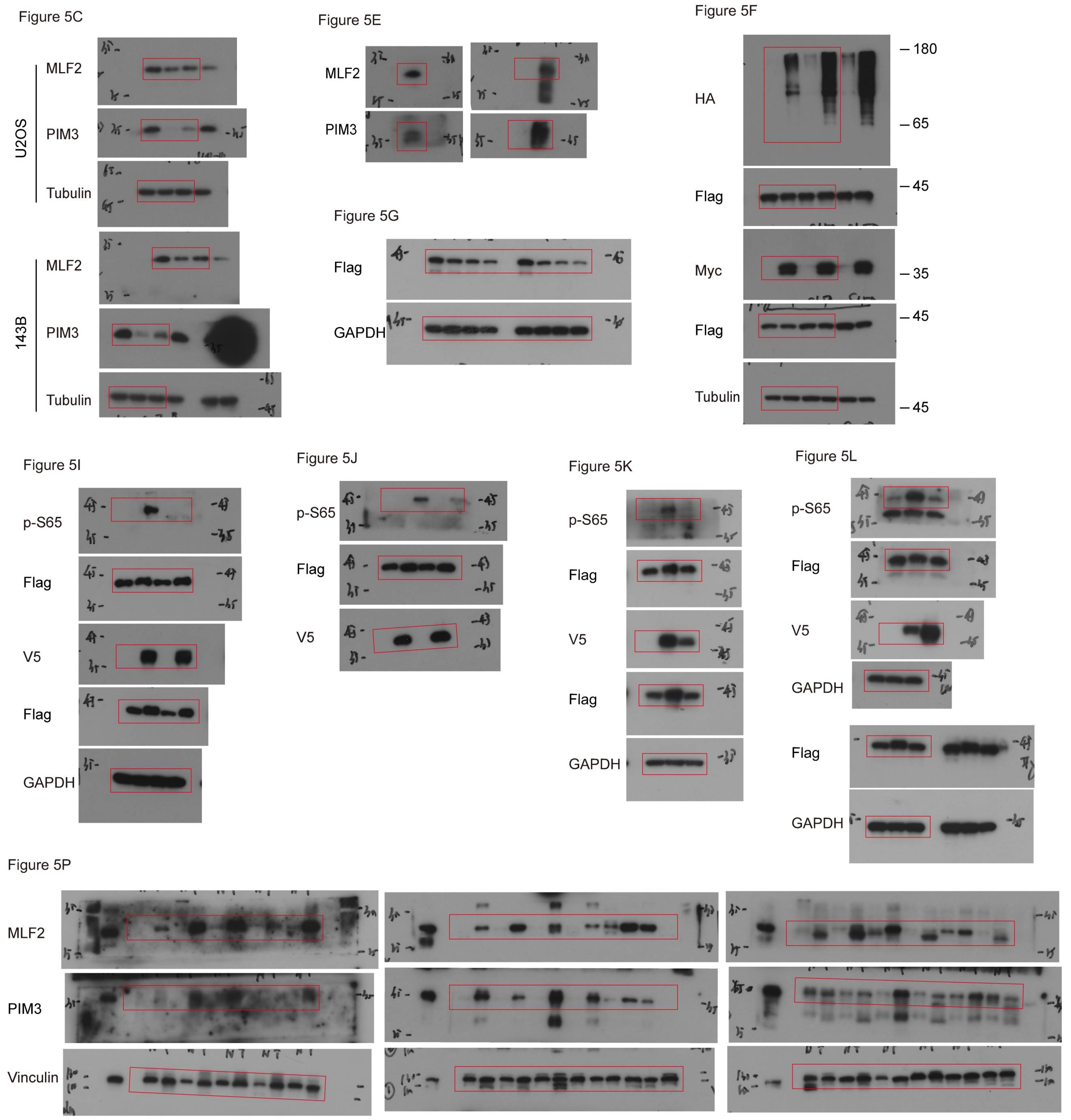

Full unedited blot for Figure6

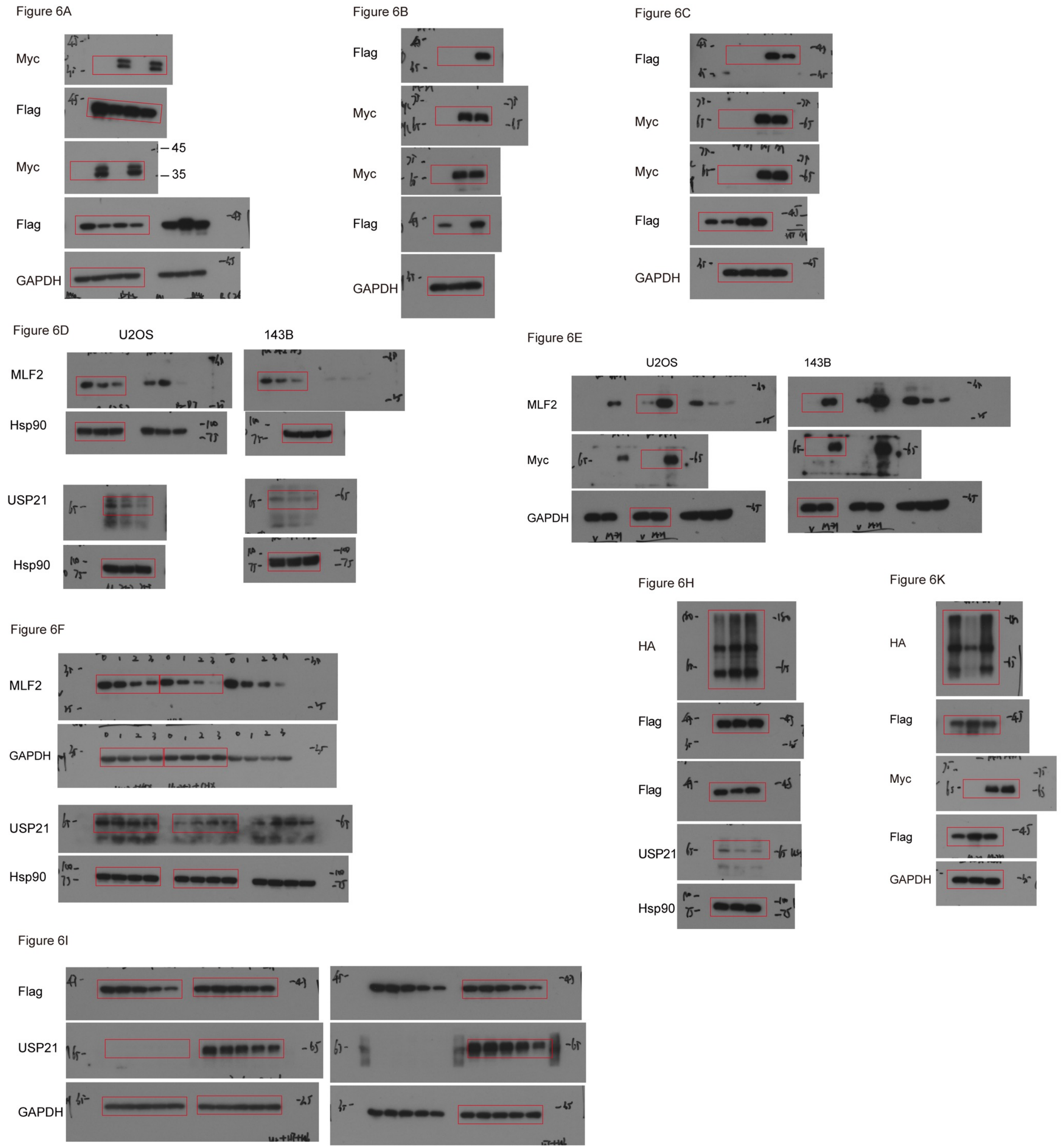

Full unedited blot for FigureS2

Supplementary Figure 2A

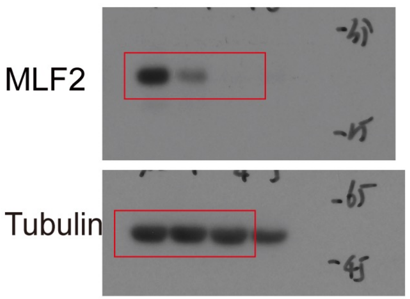

Supplementary Figure 2C

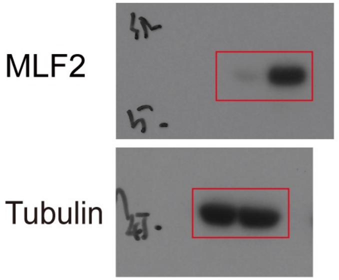

Full unedited blot for FigureS4

Supplementary Figure 4A

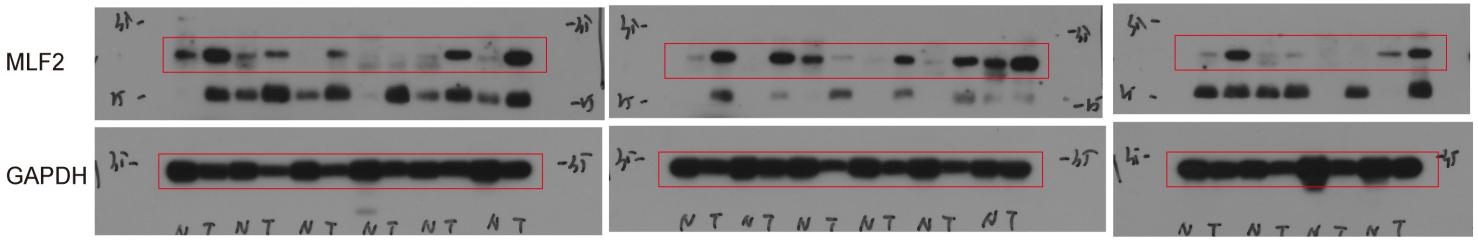

Full unedited blot for FigureS5

Supplementary Figure 5D

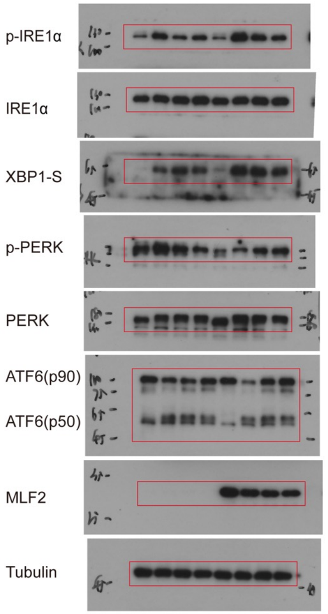

Supplementary Figure 5F

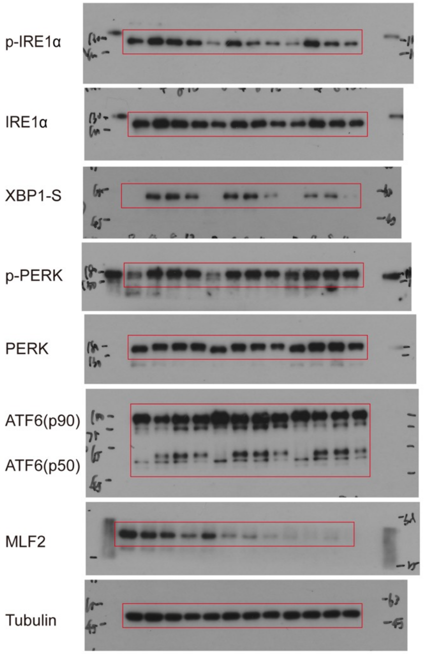

Supplementary Figure 5E

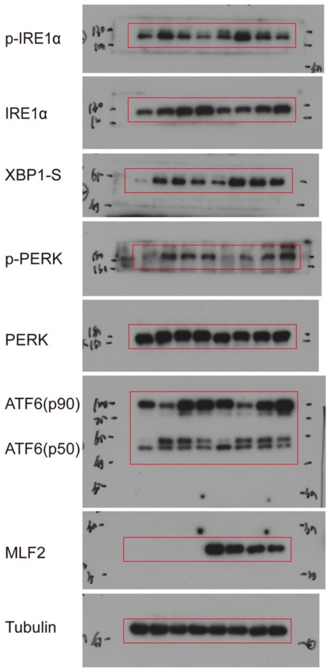

Supplementary Figure 5G

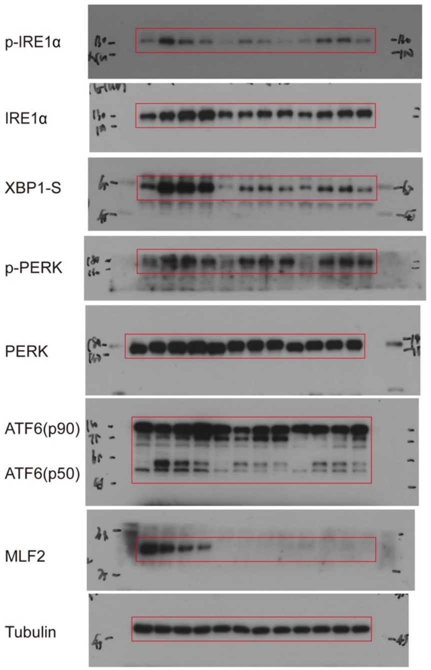

Full unedited blot for FigureS6

Supplementary Figure 6D

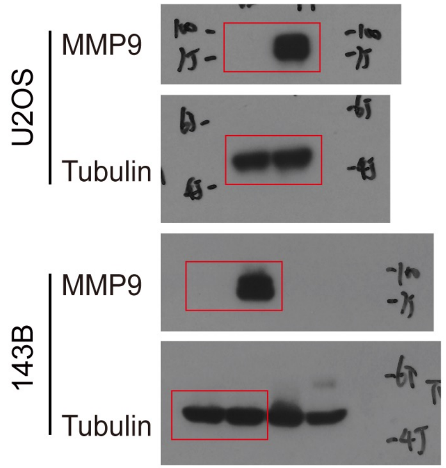

Supplementary Figure 6F

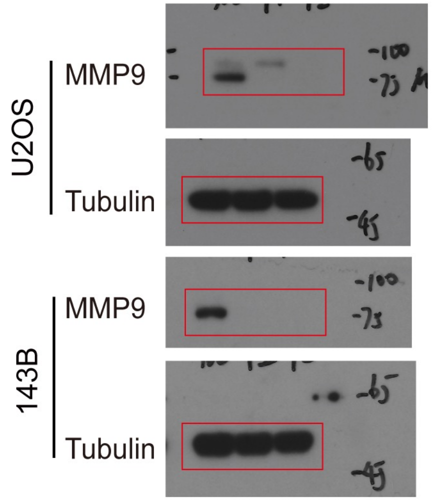

Full unedited blot for FigureS7

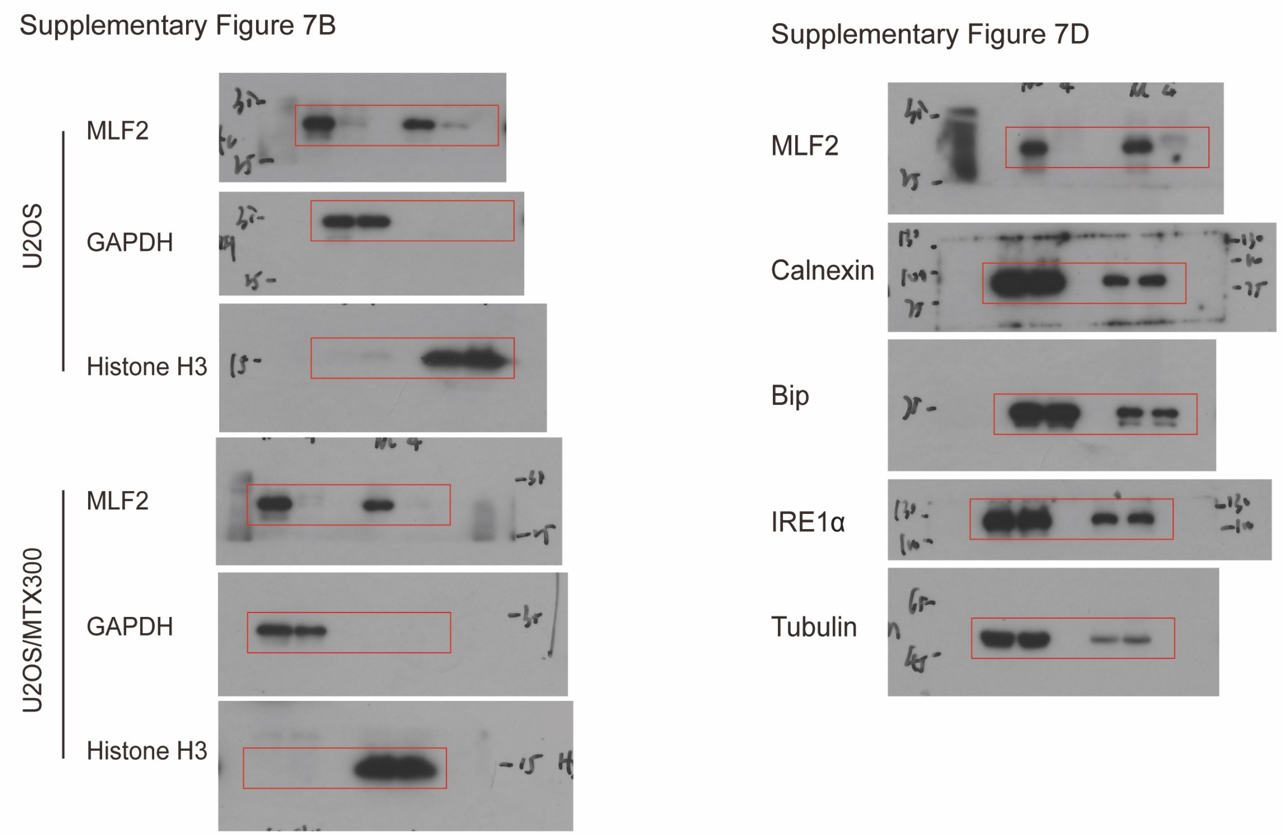

Full unedited blot for FigureS8

Supplementary Figure 8A

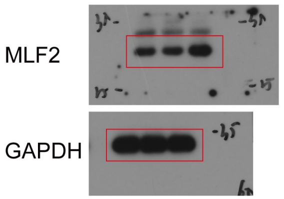

Supplementary Figure 8B

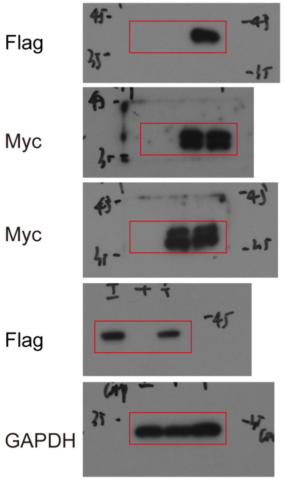

Supplementary Figure 8C

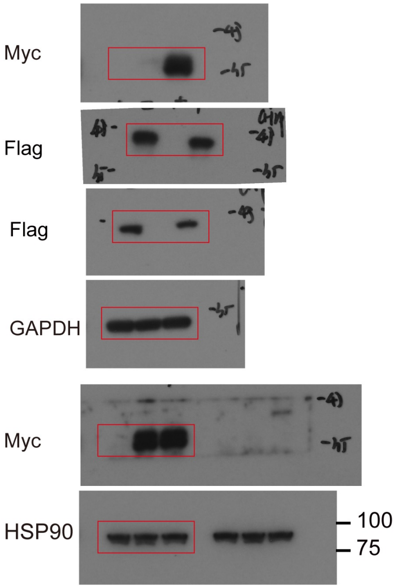

Supplementary Figure 8G

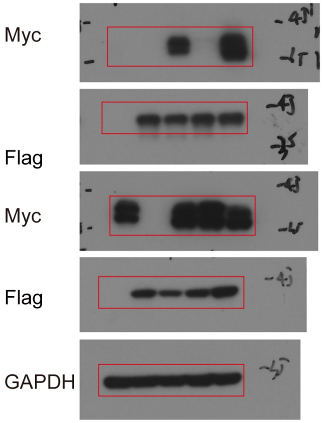

Supplementary Figure 8E

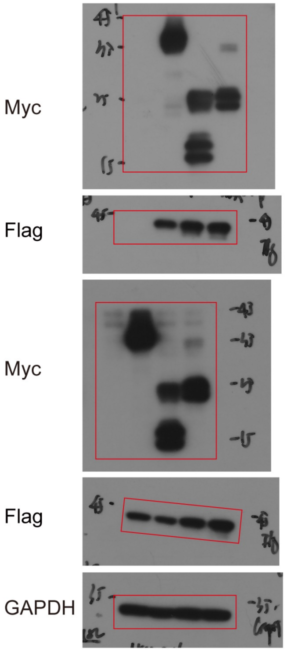

Supplementary Figure 8F

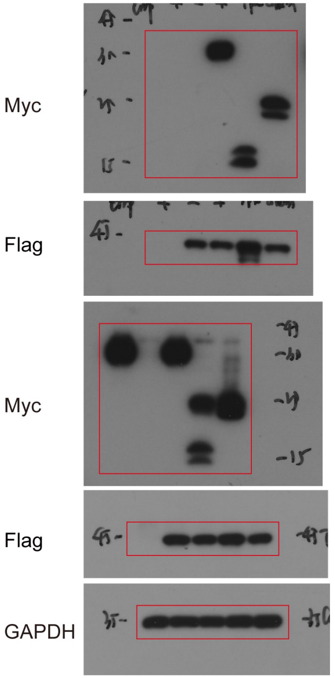

Full unedited blot for FigureS9

Supplementary Figure 9A

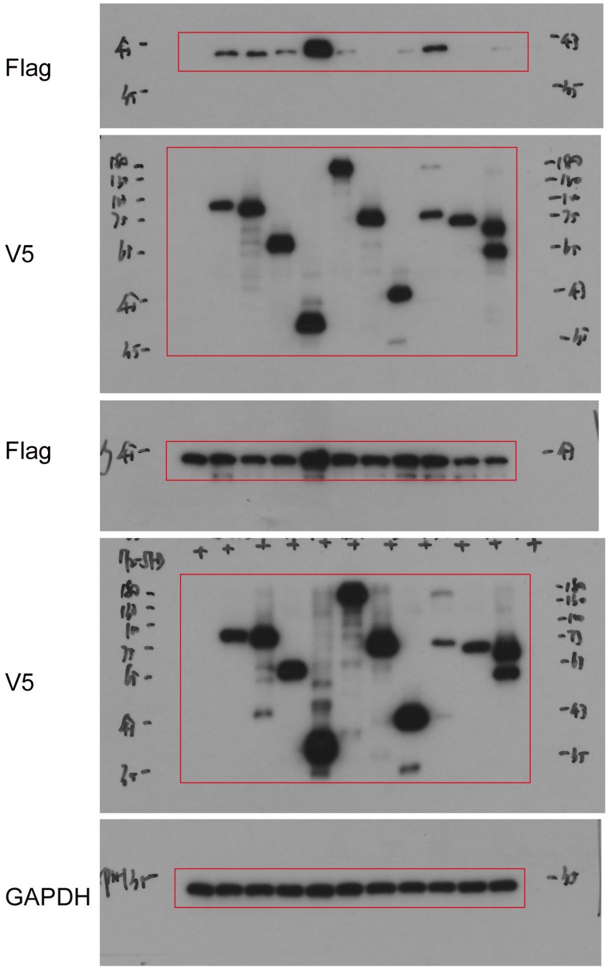

Supplementary Figure 9D

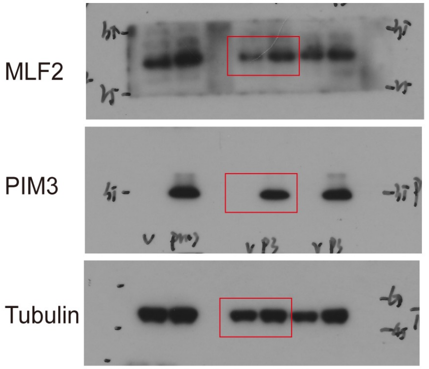

Supplementary Figure 9E

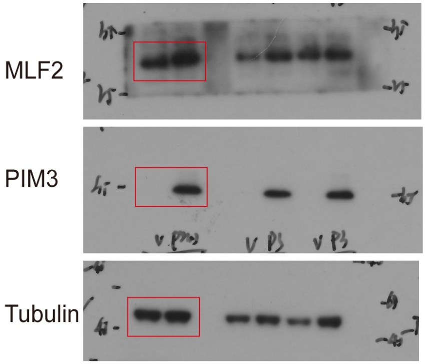

Full unedited blot for FigureS10

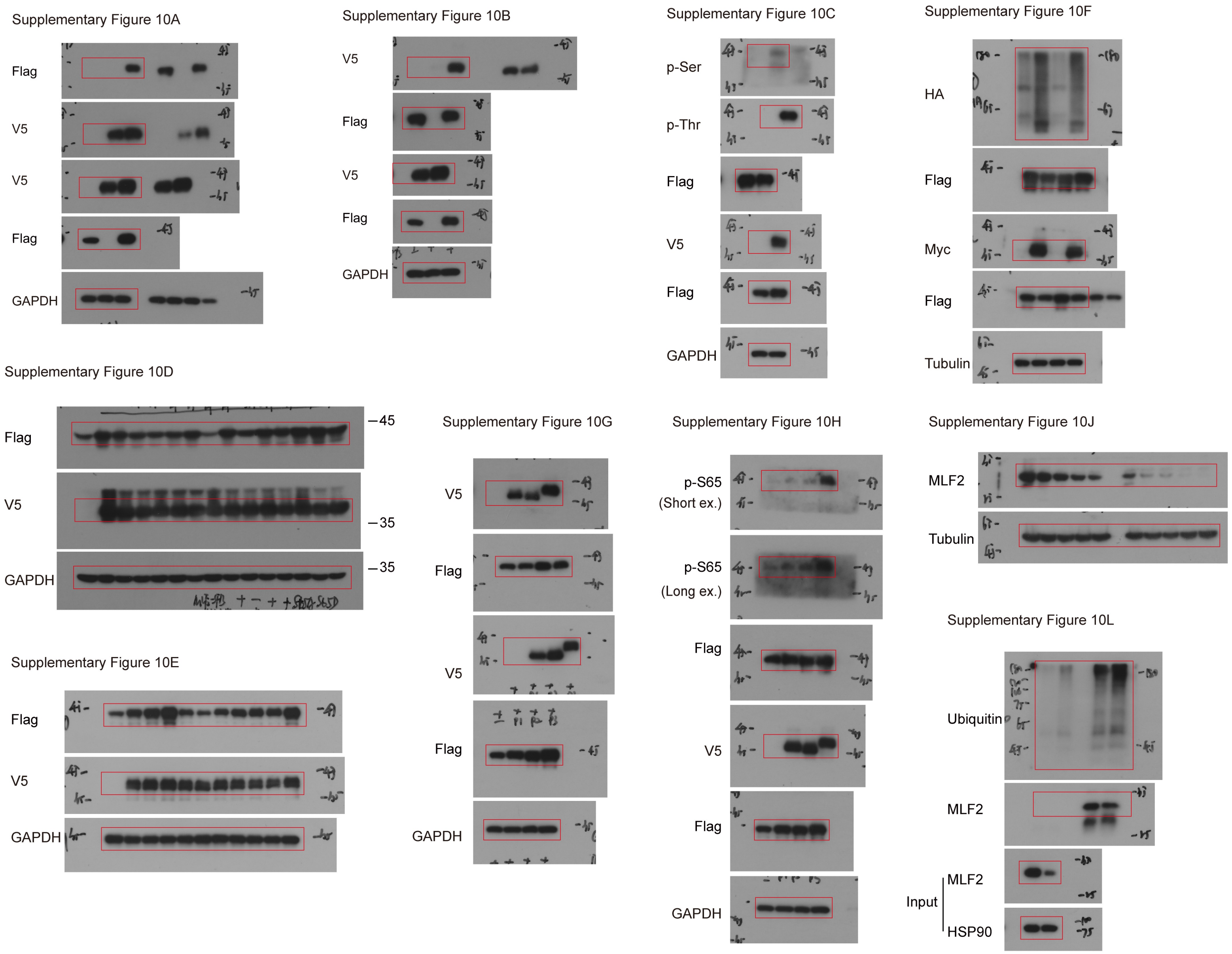

Full unedited blot for FigureS11

Supplementary Figure 11A

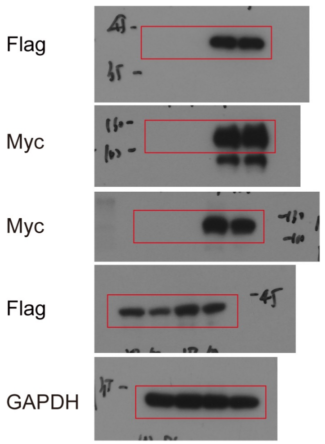

Supplementary Figure 11D

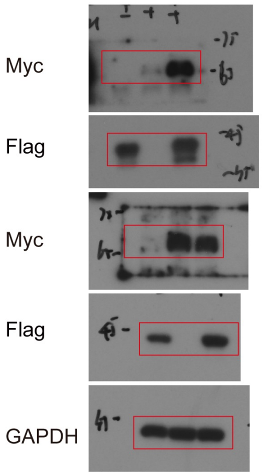

Supplementary Figure 11B

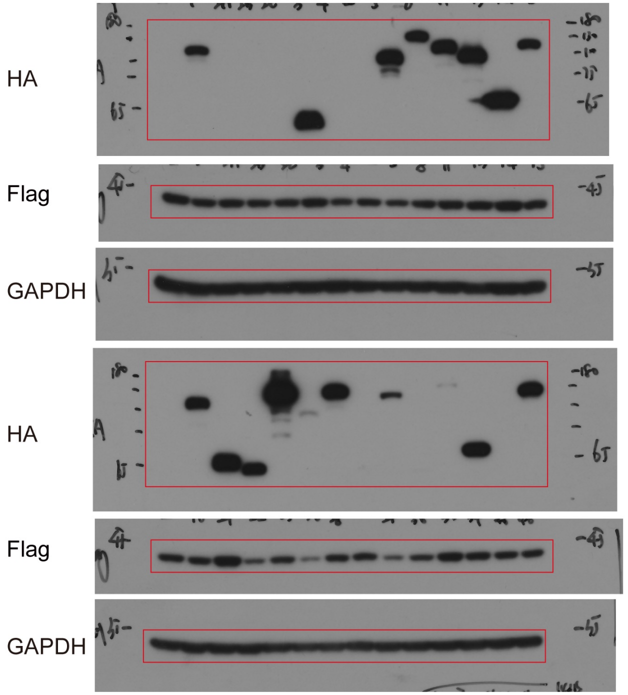

Supplement: Unedited blot and gel images [file jci-135-191040-s032.pdf]
